# Molecular Evolution of the TET Gene Family in Mammals

**DOI:** 10.3390/ijms161226110

**Published:** 2015-12-01

**Authors:** Hiromichi Akahori, Stéphane Guindon, Sumio Yoshizaki, Yoshinori Muto

**Affiliations:** 1 United Graduate School of Drug Discovery and Medical Information Sciences, Gifu University, 1-1 Yanagido, Gifu 501-1194, Japan; h.akahori@gmail.com (H.A.); s3502004@edu.gifu-u.ac.jp (S.Y.); 2 Department of Statistics, the University of Auckland, Auckland 1010, New Zealand; s.guindon@auckland.ac.nz; 3 Department of Functional Bioscience, Gifu University School of Medicine, 1-1 Yanagido, Gifu 501-1193, Japan

**Keywords:** molecular evolution, ten-eleven translocation (TET) protein, positive selection, mammal

## Abstract

Ten-eleven translocation (TET) proteins, a family of Fe^2+^- and 2-oxoglutarate-dependent dioxygenases, are involved in DNA demethylation. They also help regulate various cellular functions. Three TET paralogs have been identified (TET1, TET2, and TET3) in humans. This study focuses on the evolution of mammalian TET genes. Distinct patterns in TET1 and TET2 *vs.* TET3 were revealed by codon-based tests of positive selection. Results indicate that TET1 and TET2 genes have experienced positive selection more frequently than TET3 gene, and that the majority of codon sites evolved under strong negative selection. These findings imply that the selective pressure on TET3 may have been relaxed in several lineages during the course of evolution. Our analysis of convergent amino acid substitutions also supports the different evolutionary dynamics among TET gene subfamily members. All of the five amino acid sites that are inferred to have evolved under positive selection in the catalytic domain of TET2 are localized at the protein’s outer surface. The adaptive changes of these positively selected amino acid sites could be associated with dynamic interactions between other TET-interacting proteins, and positive selection thus appears to shift the regulatory scheme of TET enzyme function.

## 1. Introduction

DNA methylation, an important epigenetic mark of cytosine bases associated with transcriptional regulation, is known to play a crucial role in various cellular phenomena, including gametogenesis, embryonic development, and genomic imprinting [1,2]. The enzymatic processes of DNA methylation and demethylation are mediated by various specific enzymes such as DNA methyl transferases and dioxygenases, which catalyze methyl transfer or oxidative modification of bases in DNA [3,4]. The regulation of these enzymatic reactions is thought to be critical in many biological processes [5,6].

Ten-eleven translocation (TET) proteins are Fe^2+^- and 2-oxoglutarate-dependent dioxygenases. Their name “TET” reflects the ten-eleven translocation (t(10;11)(q22;q23)) that is observed in chromosomes of patients with acute myeloid leukemia or lymphocytic leukemia [7]. The metazoan TET proteins were first identified by profile searches using iterative sequences and the predicted oxygenase domains of JBP1 and JBP2a from *Trypanosoma brucei* baseJ-binding protein [8]. Further analyses identified a family of many predicted nucleic acid-modifying dioxygenases from a wide variety of eukaryotes [4,7]. Several reports have demonstrated that TET proteins are able to catalyze the oxidation of 5-methylcytosine (5mC) to 5-hydroxymethylcytosine (5hmC), 5-formylcytosine (5fC) and 5-carboxylcytosine (5caC) in DNA [7,8,9,10]. The finding that 5caC and 5fC are further excised by thymine-DNA glycosylase and substituted by unmodified cytosine [9] suggests a potential mechanism for active demethylation [7,11].

In mammals, three TET paralogs have been identified: TET1, TET2, and TET3 [4,7,11]. Each has a carboxyl-terminal catalytic core region comprising a Cys-rich domain and a double-stranded β helix (DSBH) domain with a large low-complexity insert [7,12]. The length of this low-complexity insert varies greatly among TET paralog members, and the sequence is less conserved [4]. TET1 and TET3 also have an amino-terminal CXXC domain, which was described as a DNA-binding motif [7,11]. In TET2 gene, a chromosomal inversion apparently split the ancestral TET2 gene, breaking it into distinct segments that encode the CXXC domain and the catalytic domain, which became a separate gene (IDAX) [3,4,13]. It has further been reported that the three TET proteins show different patterns of tissue-specific expression [14,15]. It has thus been speculated that TET genes diversified functionally due to adaptive evolution and gene duplication. The details of such an evolutionary process have not yet been established, however.

The present study was conducted to clarify the TET gene family’s functional differentiation and evolutionary history in mammalian species. Positive selective pressures at the gene level and the sites subjected to this regime were a focus of our investigation. We used codon models that assume that the selection patterns vary along the sequence but do not differ among lineages [16,17,18,19]. We also considered codon models that allow selection regimes to vary across sites and lineages [20]. These models revealed that the mammalian TET genes have undergone positive selection, with frequent adaptive divergence in the TET1 and TET2 genes. Our findings shed light on the evolution of TET gene family and the potential functional diversification of amino acid residues that could contribute to the regulation of various developmental processes.

## 2. Results

### 2.1. Mammalian TET Family Gene Sequences

We retrieved the available TET sequences using the Ensembl Compara database and the Blastp program. Our query of the human TET amino acid sequences in major databases helped us identify a number of homologous proteins in mammalian species. At least 39 species were available for each gene, including a broad range of mammalian species (Table S1). The wide distribution of TET1, TET2 and TET3 genes in the variety of mammalian species suggests that the TET genes underwent two successive duplications before mammalian diversification [4,7]. Since the sequence identity among the TET paralogs was relatively low other than in the catalytic domains, we analyzed each paralog separately in this study.

Anisimova *et al.* [21] showed that recombination hampers the detection of positive selection. We therefore first screened the three TET paralog alignments to determine the presence or absence of recombination breakpoints. We used the genetic algorithm recombination detection (GARD) tool implemented in the HyPhy package [22]. No evidence of recombination was supported by the Kishino-Hasegawa test (KH) with a *p*-value 0.01 for any of the three TET genes. The maximum-likelihood trees were reconstructed for TET1, TET2 and TET3 under the GTR + I + G4 nucleotide substitution model (Figures S1–S3). These phylogenetic trees were used for the subsequent evolutionary analyses.

### 2.2. Positive Selection and the Diversification of TET Family Genes

Table 1 summarizes the output of the codeml program [18,19] obtained using the site models. We used model M0, which assumes a single ω for all of codon sites in the alignment, to estimate a general ω value for each data set. The ω values were quite similar for TET1 and TET2 (ω = 0.385 for TET1 genes and ω = 0.346 for TET2 genes), while that of TET3 was distinct, with an estimated value of ω equal to 0.115. These relatively low ω values for TET genes suggest that the evolution of these genes was dominated by a type of “purifying” selection on most sites and lineages. Positive selection can be accounted for by codon models M2a and M8, by providing an additional class of sites in which ω > 1. For the TET1 and TET2 data sets, significantly better fit for the data is obtained with the M8 model (*p* < 0.001) *vs.* the corresponding null model (M7). Our comparison of M1a and M2a revealed no TET family genes under positive selection. Model M8 allows for a site-specific ω ratio that is β-distributed. This model assigned 3.8% (TET1) and 2.2% (TET2) of codon sites in the class corresponding to positive selection. The estimated values of ω under the M8 model were only slightly larger than 1, indicating that although there is evidence for significant positive selection, its intensity is moderate. The Bayes empirical Bayes approach to detect sites under positive selection indicated that two sites for TET1 and seven sites for TET2 have ω > 1 with high posterior probabilities (*i.e.*, PP > 0.95) (Table 1). Our analysis of TET3 revealed no trace of positive selection; indeed, the proportion of sites that had evolved under negative selection (*p*_0_ of the M1a model) was found to be virtually equal to one for this gene (Table 1).

**Table 1:** Likelihood analysis of the site models in the PAML program for the TET gene sequence data.

Model	Parameter Estimates ^a^	*LnL*	2Δ*lnL* (*p*-Value)	Positively Selected Sites ^b,c^
***TET1***				
M0	ω = 0.385	−87,143.158462		None
M1a	ω_0_ = 0.205, *p*_0_ = 0.623, ω_1_ = 1.000, *p*_1_ = 0.377	−86,007.766228		Not allowed
M2a	ω_0_ = 0.205, *p*_0_ = 0.623, ω_1_ = 1.000, *p*_1_ = 0.298, ω_2_ = 1.000, *p*_2_ = 0.078	−86,007.766228	*M2a vs. M1a*	None
			0 (*p* = 1)	
M7	*p* = 0.679, *q* = 0.983	−85,718.609725		Not allowed
M8	*p*_0_ = 0.962, *p* = 0.744, *q* = 1.197, *p*_1_ = 0.038, ω_1_ = 1.294	−85,707.99818	*M8 vs. M7*	524F, 811M
			21.22309 (*p* < 0.001)	
***TET2***				
M0	ω = 0.346	−75,677.716183		None
M1a	ω_0_ = 0.184, *p*_0_ = 0.664, ω_1_ = 1.000, *p*_1_ = 0.336	−74,593.700860		Not allowed
M2a	ω_0_ = 0.184, *p*_0_ = 0.664, ω_1_ = 1.000, *p*_1_ = 0.292, ω_2_ = 1.000, *p*_2_ = 0.044	−74,593.700860	*M2a vs. M1a*	None
			0 (*p* = 1)	
M7	*p* = 0.617, *q* = 1.064	−74,286.497185		Not allowed
M8	*p*_0_ = 0.978, *p* = 0.671, *q* = 1.250, *p*_1_ = 0.022, ω_1_ = 1.558	−74,265.863135	*M8 vs. M7*	123R*, 469V, 673V, 968P, 1002T, 1205S, 1767S
			41.2681 (*p* < 0.001)	
***TET3***				
M0	ω = 0.115	−48,719.653509		None
M1a	ω_0_ = 0.056, *p*_0_ = 0.860, ω_1_ = 1.000, *p*_1_ = 0.140	−47,597.069946		Not allowed
M2a	ω_0_ = 0.056, *p*_0_ = 0.859, ω_1_ = 1.000, *p*_1_ = 0.139, ω_2_ = 999.000, *p*_2_ = 0.002	−47,599.818203	*M2a vs. M1a*	None
			−5.496514 (*p* = 1)	
M7	*p* = 0.237, *q* = 1.380	−47,268.934744		Not allowed
M8	*p*_0_ = 0.999, *p* = 0.237, *q* = 1.380, *p*_1_ = 0.001, ω_1_ = 999.000	−47,270.552239	*M8 vs. M7*	None
			−3.23499 (*p* = 1)	

^a^ *p* and *q* denote the parameters of the β distribution, β (*p*, *q*), which determine that of ω values; ^b^ Amino acid sites inferred to be under positive selection are shown with a posterior probability PP > 0.95; * Posterior probability (PP) > 0.99; ^c^ The referenced sequences for the *TET1*, *TET2* and *TET3* genes were human TET1, human TET2, and human TET3, respectively.

### 2.3. Identification of Positively Selected Sites

**Figure 1:**
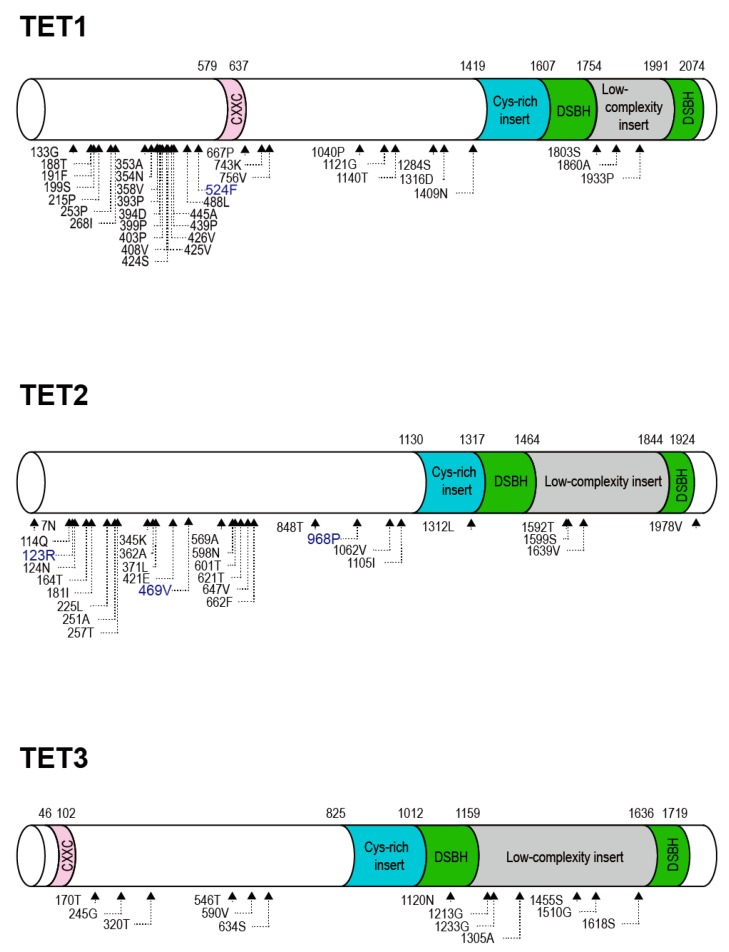
Structural representation of positively selected codon sites in mammalian TET family genes. Based on the human TET gene structure, domain diagrams of TET1, TET2 and TET3 are shown with CXXC, Cys-rich insert, DSBH and low-complexity insert domains. The corresponding domain boundaries are shown with the amino acid number above the diagram. The triangles at the bottom represent sites of positive selection identified by at least three of the six analysis methods used. Amino acid sites detected by PAML using model M8 are highlighted in blue.

We used six methods to accurately identify sites that evolved under positive selection in the TET gene families: the PAML package’s model M8 (Table 1) and the Datamonkey web server’s FEL, FUBAR, MEME, REL, and SLAC methods [23]. In accord with other studies [24,25], we considered only the codons that were identified by at least three of these methods as having undergone positive selection. As shown in Figure 1, a total of 34 codons for TET1, 29 for TET2 and 13 for TET3 were identified as positively selected. TET family proteins have a C-terminal catalytic domain that is comprised of a double-stranded β helix (DSBH, also known as the jelly-roll motif) domain with a large low-complexity insert and a Cys-rich domain (Figure 1) [12]. Interestingly, the greatest number of positively selected codons were located mostly outside the DSBH and Cys-rich domains. There were also no positively selected sites in the CXXC domain, which binds unmethylated CpG sequences [7]. This suggests that strong selective constraints are acting on this domain. In contrast, several positively selected codons were observed in the large low-complexity inserts, indicating different selective pressures acting on these regions.

### 2.4. Shifts in the Site-Specific Selection Process along Lineages

In order to clarify the processes of site-specific selection along the different lineages, we conducted maximum-likelihood analyses by employing the software program Fitmodel, using stochastic branch-site codon substitution models in a nested set [20,26]. These models are relevant in cases where no prior evidence is available about the lineages that evolved under positive selection [27]. The results of our analyses with the M0, M3, M3 + S1, and M3 + S2 substitution models are provided in Table 2 and Table 3. For the TET1, TET2, and TET3 data sets, as parameters were added to the nested substitution models, the log likelihoods were greatly improved (Table 2), suggesting that the best codon substitution model for the TET1, TET2, and TET3 data sets is the model M3 + S2—in which there are unequal switching rates among the three classes of rate ratios. Under the M3 + S2 model, the ω estimates for the three classes in the TET1 data set were ω_1_ = 0.01, ω_2_ = 0.23, and ω_3_ = 1.34 (Table 3). We observed a significantly higher switching rate between ω_2_ and ω_3_ (*R*_23_ = 3.34) compared to that between ω_1_ and ω_2_ (*R*_12_ = 0.71) and compared to that between ω_1_ and ω_3_ (*R*_13_ = 0.2). These results suggest that the changes between relatively similar selection patterns occur at a higher rate compared to the shifts that involved the most highly constrained rate ratio classes. With the M3 + S2 model, the parameter estimates indicate that most of the sites (74%, corresponding to *p*_1_ + *p*_2_) have undergone purifying selection (with ω < 1 in the TET1 data set), and 25% (*p*_3_) of the sites within the TET1 gene underwent positive selection (with a ω value >1). This pattern of parameter estimates resembles that inferred for the TET2 data set (Table 3). For the TET3 data set, however, 9% of the codons are thought to have undergone relaxed selection (ω_3_ = 1.07) with the M3 + S2 model (Table 3). The majority of codon positions in TET3 are thought to have undergone strong purifying selection; the equilibrium frequency of sites in the ω class was 68% for ω_1_ = 0.003 and 23% for ω_2_ = 0.22 respectively (Table 3).

**Table 2:** Likelihood ratio tests (LRTs) between different model comparisons in the Fitmodel program.

Gene	M0 *vs.* M3		M3 *vs.* M3 + S1		M3 + S1 *vs.* M3 + S2	
	2Δ*lnL*	*p*-Value	2Δ*lnL*	*p*-Value	2Δ*lnL*	*p*-Value
***TET1***	2858.77	<0.001	376.92	<0.001	71.11	<0.001
***TET2***	2803.21	<0.001	345.29	<0.001	63.69	<0.001
***TET3***	2926.57	<0.001	160.22	<0.001	12.59	<0.002

**Table 3:** Likelihood analysis of the models in the Fitmodel program for the TET gene sequence data.

Parameter	M0	M3			M3 + S1			M3 + S2		
***TET1***										
*L*n*L*	−87,135.73	−85,706.34			−85,517.88			−85,482.33		
ω_1_ ω_2_ ω_3_	0.39	0.04	0.35	0.90	0.00	0.42	1.21	0.01	0.23	1.34
*p*_1_ *p*_2_ *p*_3_		0.25	0.48	0.26	0.37	0.41	0.22	0.28	0.46	0.25
*R*_12_ *R*_13_ *R*_23_					1.54	1.54	1.54	0.71	0.2	3.34
***TET2***										
*L*n*L*	−75,677.72	−74,276.11			−74,103.47			−74,071.62		
ω_1_ ω_2_ ω_3_	0.35	0.04	0.36	0.94	0.00	0.42	1.24	0.005	0.30	1.54
*p*_1_ *p*_2_ *p*_3_		0.31	0.50	0.19	0.39	0.44	0.17	0.35	0.48	0.17
*R*_12_ *R*_13_ *R*_23_					1.6	1.6	1.6	0.74	0.33	4.34
***TET3***										
*L*n*L*	−48,719.65	−47,256.37			−47,176.26			−47,169.96		
ω_1_ ω_2_ ω_3_	0.11	0.01	0.19	0.75	0.00	0.28	0.98	0.003	0.22	1.07
*p*_1_ *p*_2_ *p*_3_		0.62	0.28	0.10	0.69	0.23	0.08	0.68	0.23	0.09
*R*_12_ *R*_13_ *R*_23_					2.14	2.14	2.14	1.04	2.27	9.96

**Figure 2:**
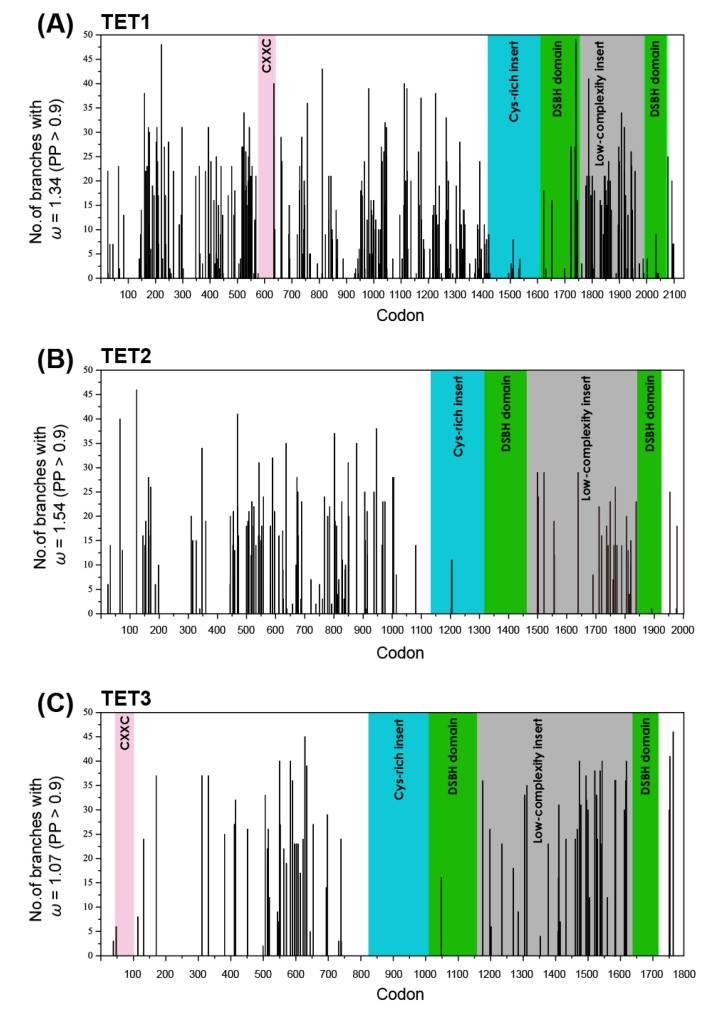
Distribution of branches with the ω_3_ rate ratio class on each site across the TET1 (**A**); TET2 (**B**) and TET3 (**C**) alignments. The CXXC, Cys-rich insert, DSBH and low-complexity insert domains are in color. The *x*-axis represents the position of codons in the alignment, and the *y*-axis represents the number of branches exhibiting the ω_3_ rate ratio class (PP > 0.9).

We next evaluated the number of branches showing traces of positive selection at each site of the TET gene alignments. The TET1 alignment included 2114 codons, of which 298 (14%) revealed evidence of moderate positive selection (ω_3_ = 1.34) which would have taken place at some time in the TET1 gene family’s history (PP > 0.9; Figure 2A). For the TET2 alignment which included 1905 codons, 128 (6.7%) sites were inferred to have ω_3_ = 1.54 at some point in the history with high posterior probabilities (Figure 2B). As is the case with several site models described above (Figure 1), evidence of positive selection of one or more branches was observed for only a few codon positions in the Cys-rich, DSBH and CXXC domains (Figure 2A,B). In contrast, moderate positive selection was inferred on several branches for many codon sites in the N-terminal half and low-complexity insert regions, respectively (Figure 2A,B). We inferred that 77 (4.3%) sites in the TET3 dataset had undergone relaxed selection (ω_3_ = 1.07) at some stage in the TET3 gene family’s history, with high posterior probabilities. The TET3 codon positions for which we observed evidence of relaxed selection (ω_3_) were also distributed mainly outside the Cys-rich, DSBH and CXXC domains (Figure 2C).

Among the TET gene tree branches, the number of sites in the ω_3_ rate ratio class showing high PPs on each branch varied from 0 to 168 for TET1, from 0 to 107 for TET2 and from 0 to 64 for TET3 (Figure S4). The branches with a greater number of sites in the ω_3_ rate ratio class were concentrated on deep, internal branches of the TET1, TET2 and TET3 gene trees (Figures S1–S3).

### 2.5. 3D Structure Mapping

**Figure 3:**
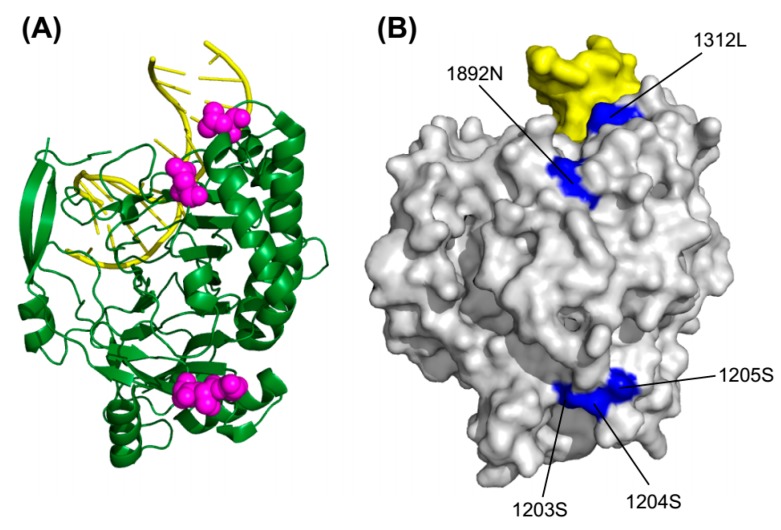
Sites under positive selection in the 3D structure of human TET2 catalytic core domain. (**A**) Ribbon representation of the human TET2 catalytic domain in complex with DNA duplex. The DNA is colored yellow. Five sites under positive selection are highlighted in the predicted structure with their side chains shown (magenta spheres); (**B**) Molecular surface representation of the human TET2 catalytic domain. The same five sites under positive selection are distributed on the molecular surface (accented in blue). The representations are in the same orientation.

We next sought to clarify the roles that positive selection played. We created a map of the positively selected amino acid sites, on a three-dimensional (3D) model of the TET protein. Since the crystal structure has been elucidated only for the catalytic core region of TET2, we focused on the 3D model of this structure. Figure 3 shows the 3D structures of the catalytic region of TET2 (PDB ID: 4NM6), which was based on the crystal structure of a catalytically active form of human TET2 bound to DNA [12]. Based on the results of our analysis using the Fitmodel program, we inferred that only four sites in the TET2 catalytic core region had ω > 1 with high PP values (>0.90): 1203S, 1204S, 1205S and 1892N. Under the site models in the PAML and Datamonkey web server, we also deduced that one site, 1312L, had undergone positive selection. We mapped these five positively selected sites on the TET2 catalytic core region’s 3D structure (Figure 3A). Four of the five amino acids identified as evolving under positive selection were in the Cys-rich domain of the catalytic core region; the other single positively selected amino-acid residue was in the DSBH domain. As clearly indicated by the molecular surface representation (Figure 3B), all five of the amino acids that appeared to have undergone positive selection were on the outer surface of the protein.

### 2.6. Convergent and Divergent Changes

**Figure 4:**
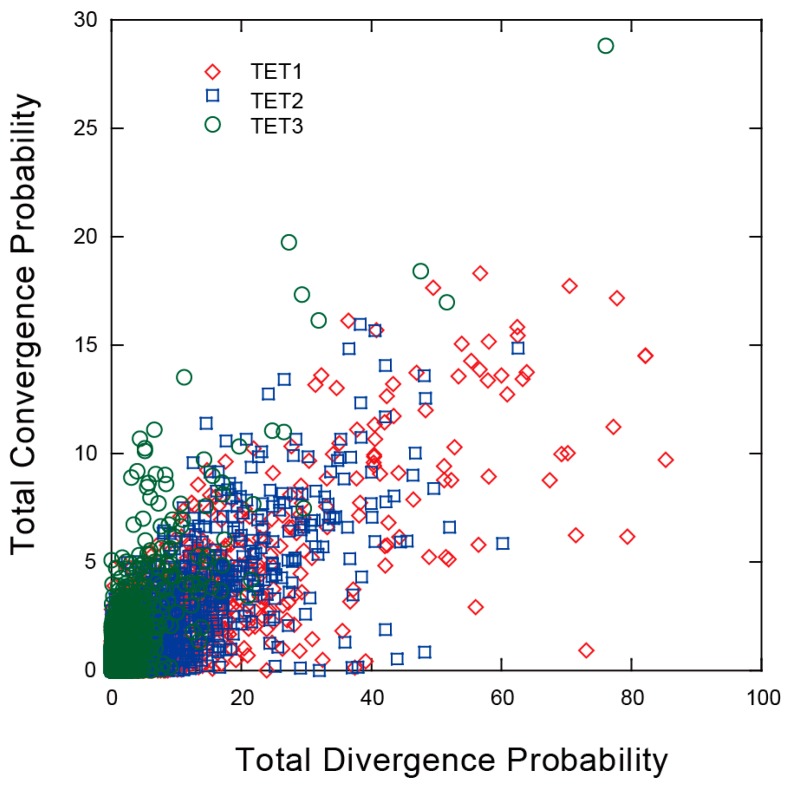
Total posterior probabilities of divergence *vs.* total posterior probabilities of convergence for all pairs of branches in the TET gene tree. TET1 pair-wise branch comparisons (red), TET2 pair-wise branch comparisons (blue) and TET3 pair-wise branch comparisons (green).

Using the codeMLancestral program [28], we attempted to identify the parallel and convergent amino-acid substitutions, based on branch-wise comparisons of the TET genes. Figure 4 shows the plots of the summed PP values of total convergent substitutions (that is, both the convergent and parallel substitutions) *vs.* the summed PP values of total divergent substitutions across all of the branch-pair comparisons for the TET1, TET2 and TET3 phylogenetic trees. Similar to other studies [28,29], there was a linear relationship between the number of divergent and convergent substitutions, and the divergent substitutions were more abundant than convergent substitutions. In the case of TET3, although these relationships were noticeable, we also observed extremely lower divergence probabilities and somewhat higher convergence probabilities compared to TET1 and TET2. These results suggest that TET3 experienced different evolutionary dynamics during mammalian evolution.

## 3. Discussion

TET family genes are implicated in the control of various aspects of cellular differentiation and transformation, and their expression levels in a variety of cell types and tissues were thus observed to differ [15,30,31]. The expressions of both TET1 and TET2 in mouse embryonic stem (ES) cells are high. TET3, in contrast, is highly enriched in the mouse oocyte and early zygote. During the zygotic stage of early embryonic development, the male pronuclei experience a mass cytosine oxidation that is mediated by TET3 [31]. At the blastocyst stage, the inner cell mass (that gives rise to the ES cells), undergoes extensive DNA methylation. At this stage, TET1 and TET2 are highly expressed, probably reconstructing and adjusting methylation patterns [7]. These different expression patterns among TET paralogs further indicate that these proteins have distinct significant biological functions in developmental processes and in various tissues [31]. We conducted an evolutionary analysis of the three TET paralogs in the present study, to clarify the role of selective pressure in the functional divergence of this gene family.

We found that for the mammalian TET genes, the predominant selective regime was negative selection, based on estimates of the nonsyonymous to synonymous rate ratio obtained under model M0. This result highlights the functional importance of TET genes in the physiology and survival of mammals [32]. We also observed that the site models—which characterize the selection pattern at the levels of sites—disclosed a number of codon sites that showed evidence of positive selection. We next attempted to clarify whether this evidence of positive selection acted equally in all subfamilies or was restricted to specific to subfamilies. According to model M8, TET1 and TET2 appeared to have undergone positive selection that acted on a subset of sites, whereas none of the site models implemented in PAML revealed positive selection within TET3. We then used the program Fitmodel to examine the potential differences in the site-specific selection patterns among the various branches in the TET phylogenies. Fitmodel uses a maximum-likelihood phylogeny-based codon substitution model with parameters for switching between the selection processes across the phylogeny and along sites [20,27]. The results obtained with Fitmodel, similar to those of the codeml analysis using site models, suggested that purifying selection played an important role in the evolution of TET genes across the greater part of their evolutionary history. However, our present findings indicate that branch- and site-specific shifts in selection occurred in each of the TET subfamilies. TET1 and TET2 displayed many sites under positive selection along some branches in the evolutionary history, suggesting that TET1 and TET2 genes evolved rapidly, which probably resulted in the functional divergence. However, we did not observe any evidence of positive selection for any branches or any sites in the TET3 subfamily with the use of the Fitmodel program, similarly to the PAML site models. The evolutionary modes underlying the three subfamilies thus differ; in TET3 the sequences are relatively conserved, and in TET1 and TET2 they are more variable. This difference in evolutionary modes could well have played a role in their functional divergence. The results obtained in the analysis for convergent amino acid substitutions (Figure 4) also support the different evolutionary dynamics among TET gene subfamily members.

The catalytic domain of TET protein is located in the carboxyl-terminal region and recognizes 5mC in a CpG context for enzymatic oxidation. Previous structural and mutagenesis studies of TET protein [4,12] revealed the amino acid residues essential for enzymatic activity and cofactor orientation. Our analysis using the Fitmodel program and the site models in the PAML and Datamonkey programs identified five codon positions with significantly elevated ω values in the TET2 catalytic domain. These findings indicate that these positions could be especially important for the adaptive processes. Interestingly, we observed no overlap between the amino-acid residues that were proposed to be crucial for catalytic activity and the positively selected sites. This is expected because these residues are probably highly constrained as part of the dioxygenase function of TET2 protein. However, it is important to note that all of the amino-acid sites identified as having undergone positive selection in the catalytic domain are in the protein’s outer surface. We speculate that these positive-selection patterns imply that selection is probably acting on the enzyme surface (which interacts with other TET-interacting proteins), and thus positive selection appears to shift the regulatory scheme of TET enzyme function. We also compared the positively selected sites to the human UniProt entries to obtain natural variant information. Some of the positively selected sites were found to be located at, or in close proximity to a natural cancer variant. For example, positions 123, 817, 1204, 1762 and 1811 of TET2 were inferred to be have undergone positive selection by the Fitmodel software program and located at natural variants found in myeloproliferative neoplasms [33]. At present, however, it remains unclear whether this observation is statistically significant and how positively selected sites function in the TET protein.

## 4. Experimental Section

### 4.1. Sequence Retrieval and Phylogenetic Reconstruction

All homologous TET sequences were retrieved essentially as described in [26]. We first collected the mammalian TET paralogs and orthologs from the GenBank (http://www.ncbi.nlm.nih.gov/genbank/) non-redundant protein database, using the human TET1, TET2 and TET3 amino acid sequences as the query and conducting separate searches with Blastp (http://blast.ncbi.nlm.nih.gov/Blast.cgi) [34]. For a further analysis, we retrieved the nucleotide and predicted amino acid sequences. We also downloaded orthologs for human TET1, TET2 and TET3 (using the Ensembl API v80 program, http://www.ensembl.org/info/docs/api/index.html) from the Ensembl Compara database (http://www.ensembl.org/info/genome/compara/index.html) [35]. We then screened the data sets we obtained in order to delete the redundant sequences, incomplete sequences, and any sequences with ambiguous base calls. Thus, for the final analyses, 130 mammalian sequences were examined. Table S1 lists the species and sequence accession numbers.

We used the MAFFT program (http://mafft.cbrc.jp/alignment/software/) to align amino acid sequences along with parameter settings that were optimized for the iterative refinement method (FFT-NS-i) [36]. We trimmed the resulting multiple sequence alignment by deleting the poorly aligned regions (using trimAl 1.2 with the option “automated1”) [37]. A codon alignment was generated from the multiple sequence alignment of amino acid sequences (Figures S5–S7) and the corresponding DNA sequences with the use of the PAL2NAL program [38]. We used PhyML v3.0 (https://code.google.com/p/phyml/) [39] to build maximum likelihood trees after we identified the optimal model of sequence substitution by using jModeltest 2 (https://github.com/ddarriba/jmodeltest2) [40]. We used the approximate likelihood ratio (aLRT) Shimodaira-Hasegawa (SH)-like method to determine branch support, and a general time-reversible model with invariant sites and gamma values distributed as an among-site rate variation that had four rate classes (*i.e.*, GTR + I + G4), selected using the Akaike information criterion.

### 4.2. Recombination Detection

We used the HYPHY package’s GARD algorithm [22] to test for the presence of recombinations between sequences in the alignment of TET genes. The GARD algorithm uses phylogenetic incongruence among fragments of a sequence alignment to detect recombination events with the application of a genetic algorithm which allows searching for multiple breakpoints. We executed the GARD analysis using the general reversible model (GTR), and we employed a general discrete distribution that has four rate classes. We performed the Kishino-Hasegawa test [41] as it is used in the GARDProcess.bf batch file of the HYPHY package, as described [42] to identify the statistically significant recombination breakpoints.

### 4.3. Molecular Evolutionary Analysis

We used a maximum likelihood approach to identify traces of positive selection and detect the amino acid sites that evolved under positive selection. The codeml program in the PAML package ver. 4.8 [43] and the Fitmodel program ver. 0.5.3 as described in [20] were used to this end. We performed the codeml analysis separately for the TET1, TET2 and TET3 alignments. We used site models that allow the ω ratio to vary among sites [18,19]. To investigate the significance of positive selection between each pair of models (*i.e.*, M1a *vs.* M2a and M7 *vs.* M8), we conducted likelihood ratio tests (LRTs) that employed a χ^2^ distribution with two degrees of freedom. In the first pair of models, the null model M1a provides two codon site categories with corresponding proportions *p*_0_ and *p*_1_, with ω_0_ < 1 and ω_1_ = 1 respectively. Here, ω = dN/dS; dN represents the non-synonymous substitution rates, and dS represents the synonymous substitution rates. M2a, the alternative model, has an additional category for codons, with frequency *p*_2_ and ω_2_ > 1, which indicates positive selection. M7 and M8 is the second set of site models compared. M7 specified a null model that has a β distribution, with β (*p*, *q*), of ω values between 0 and 1. Model M8 is an alternative model that provides another category for sites that have ω-values >1, which indicates positive selection.

The LRT statistic is equivalent to two times the difference between the scores of the log likelihood for the two models being compared (2Δln*L* = 2(ln*L*_1_ − ln*L*_0_)). Provided that the null hypothesis is correct, the distribution of LRT statistic is approximately that of a χ^2^ distribution with two degrees of freedom. Tests that yielded negative LRT values were re-run several times using different initial ω values (0.05, 0.1 and 0.15), and the maximum log-likelihood scores obtained for each model were used to calculate the LRT. We obtained the χ^2^ critical values by using the PAML package’s χ^2^ program. We used the Bayes Empirical Bayes approach to determine the posterior probability (PP) that a given codon evolved under positive selection in the cases of alternative models that allow for positive selection [44]. The PP cut-off value of >0.95 was used to identify the sites that were under positive selection.

We also applied five other maximum likelihood methods provided in the Datamonkey web server [23]: the Fixed Effect Likelihood (FEL) method, the Fast Unbiased Bayesian AppRoximation (FUBAR) [17] method, the Mixed Effects Model of Evolution (MEME) method [45], the Single Likelihood Ancestor Counting (SLAC) method, and the Random Effect Likelihood (REL) method [16]. As the criteria to identify codons under positive selection, we used a posterior probability value >0.90 for the FUBAR method, the cut-off *p*-value of 0.1 for the FEL, MEME and SLAC methods, and the Bayes Factor >50 for the REL method.

For the separate analyses of the TET1, TET2 and TET3 alignments, we applied the Fitmodel software program, which uses Markov-modulated Markov models of codon evolution [20], or switching models. The switching models allow variations in the site-specific selection process along lineages of a phylogeny. For the present analysis we used the M0, M3, M3 + S1, and M3 + S2 models. In the M0 model, it is assumed that all of the sites have the same ω values. In the M3 model, it is assumed that there are three differing ω values (ω_1_ < ω_2_ < ω_3_). If the switching rates between ω values (ω_1_ to ω_2_, ω_1_ to ω_3_ and ω_2_ to ω_3_) are assumed to be equal, the switching model is designated as S1; if these values are not assumed to be equal, the switching model is designated as S2. We conducted LRTs between each pair of nested models (*i.e.*, M0 *vs.* M3, M3 *vs.* M3 + S1, and M3 + S1 *vs.* M3 + S2). A χ^2^ distribution was used to determine the significance of differences in the log-likelihoods. We evaluated the PP values of the third selection class (corresponding to ω_3_) at each codon position and each branch by using the Fitmodel program, and the ouput was processed using a custom script in Perl. Natural variant information of sites under positive selection for each protein was obtained from UniProt Database [46].

### 4.4. Quantifying the Convergent Amino Acid Substitutions and Divergent Amino Acid Substitutions

We used the codeMLancestral package [28] and the Castoe *et al.* [28] method to estimate the convergent and divergent amino acid substitutions. Using a JTT amino acid substitution model, we estimated the site-wise PP values of all of the possible convergent and divergent amino acid substitutions along each branch of the TET gene tree. The branch lengths of the TET gene tree were estimated using the PhyML v3.0 software. To determine the convergence and divergence probabilities for all pairwise branch comparisons, we summed the probabilities of the convergent substitutions and the divergent substitutions for each amino acid site in the branch pair by using a custom script in Perl. We plotted the total PP values of convergence *vs.* the total PP values of divergence for all of the pairs of branches in the tree.

### 4.5. Protein Tertiary Structure Visualizations

We used the PyMOL Molecular Graphics System, ver. 1.3 (Schrödinger, New York, NY, USA) to visualize the 3D structure of the TET catalytic domain and to map the positively selected sites onto the 3D structure.

## 5. Conclusions

In conclusion, we identified TET gene family members in a wide range of mammalian species, and our evolutionary analyses clarified the genes’ functional significance. Our findings indicate that the dominant force acting on these genes was purifying selection, but we also observed signs that suggest both functional divergence and adaptive evolution. We found that TET1 and TET2 genes underwent positive selection more frequently than TET3 gene, possibly due to changing selective constraints during their evolutionary process. Our examination of the positively selected sites in the catalytic domain of the TET2 protein revealed five positively selected sites on the protein’s surface. Further analyses are necessary to test our hypothesis that the TET catalytic domains underwent positive selection, changing the genes’ regulatory properties. Our results suggest however that these domains acquired several unique molecular-level modifications that enabled the TET genes to adapt and regulate the various cellular functions operative in the diverse mammalian lineages.

## Supplementary Materials

Supplementary materials can be found at http://www.mdpi.com/1422-0067/16/12/26110/s1.
