# Supplementary material for: Molecular Evolution of the TET Gene Family in Mammals
Source: Int J Mol Sci. 2015 Dec 1;16(12):28472–85. doi: 10.3390/ijms161226110 (PMC4691057; doi:10.3390/ijms161226110)
Supplement: Supplementary file 1 [file ijms-16-26110-s001.pdf]

Supplementary Materials: Molecular Evolution of the TET Gene Family in Mammals

Hiromichi Akahori, Stéphane Guindon, Sumio Yoshizaki and Yoshinori Muto

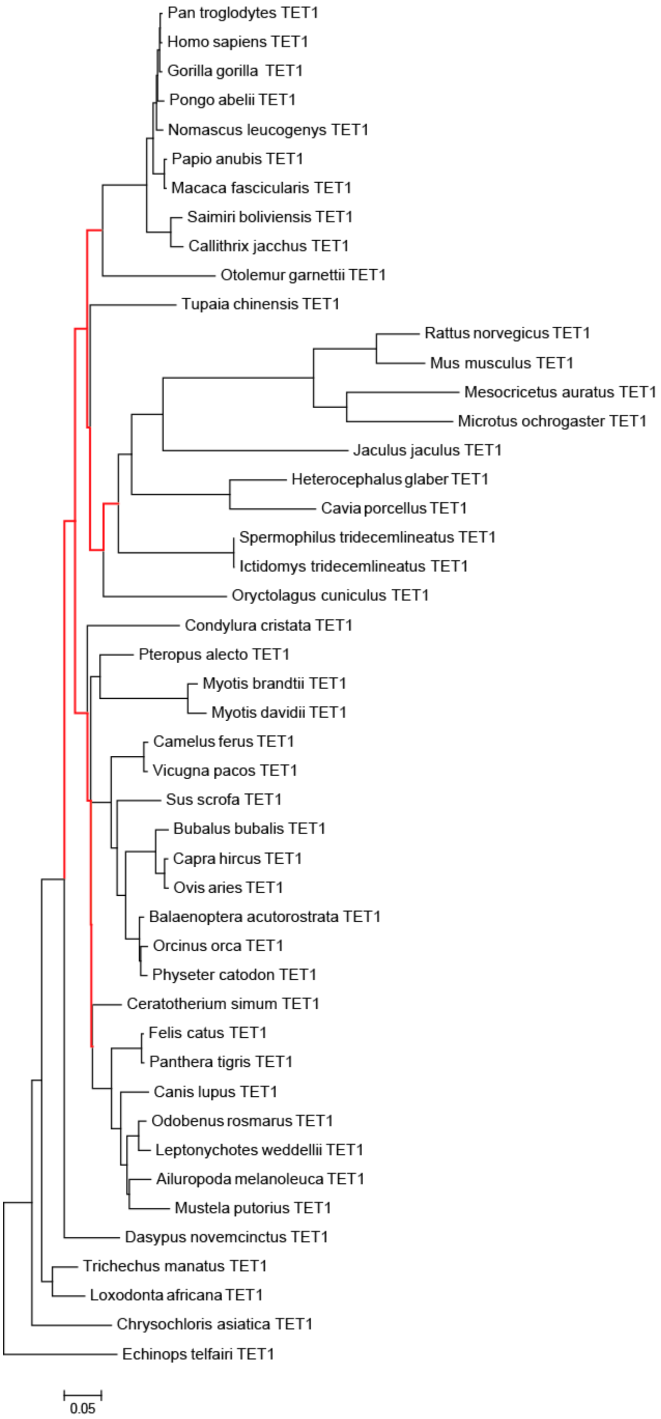

Figure S1. Maximum-likelihood tree of the TET1 genes. The tree was constructed using PhyML v3.0 software, based on the nucleotide-coding sequences. Branches with the number of sites under positive selection (ω_3 = 1.34) greater than the 90th percentile in the Fitmodel analysis (Figure S4) are shown in red.

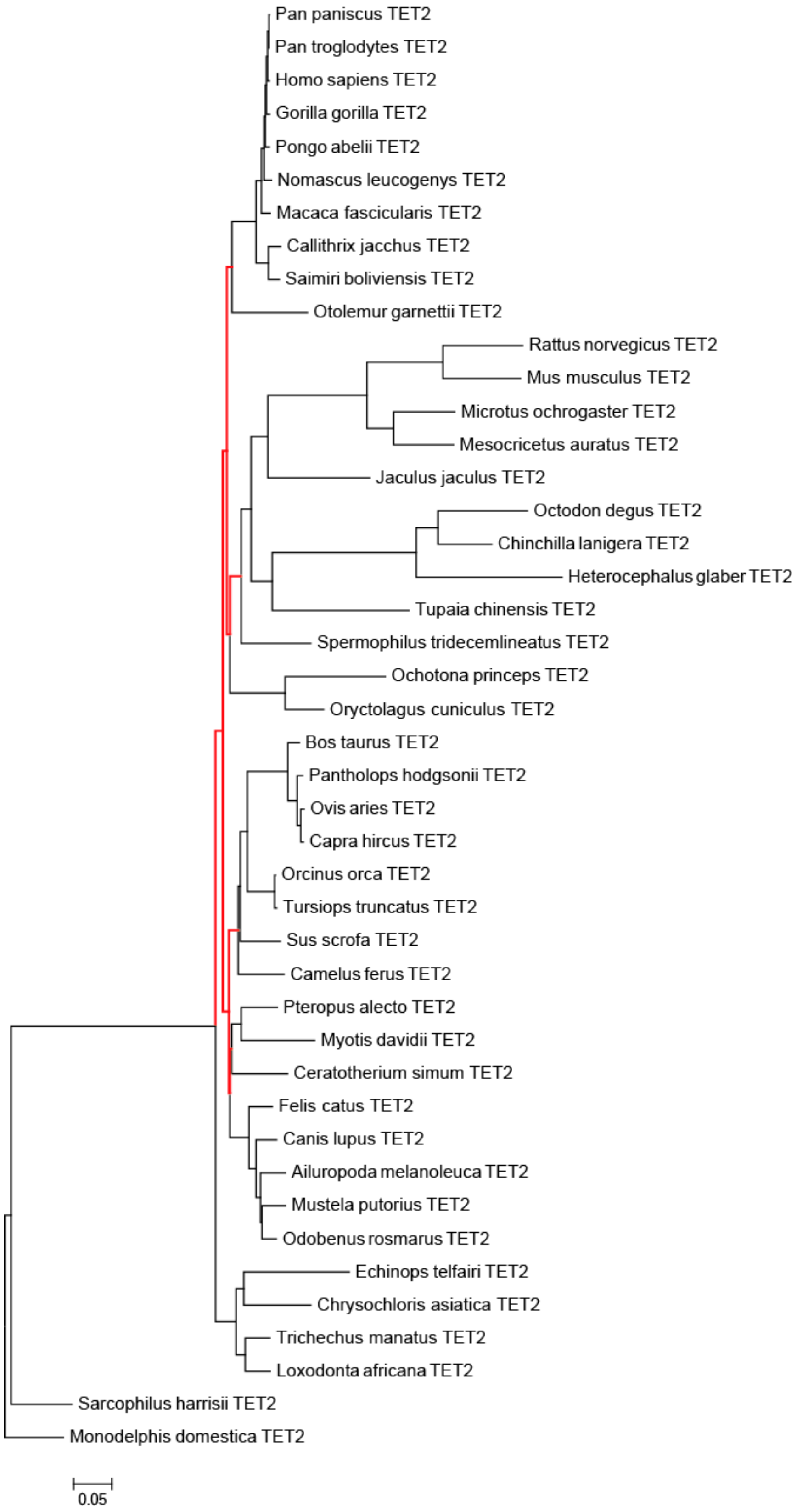

Figure S2. Maximum-likelihood tree of the TET2 genes. The tree was constructed using PhyML v3.0 software, based on the nucleotide-coding sequences. Branches with the number of sites under positive selection (ω_3 = 1.54) greater than 90th percentile in the Fitmodel analysis (Figure S4) are shown in red.

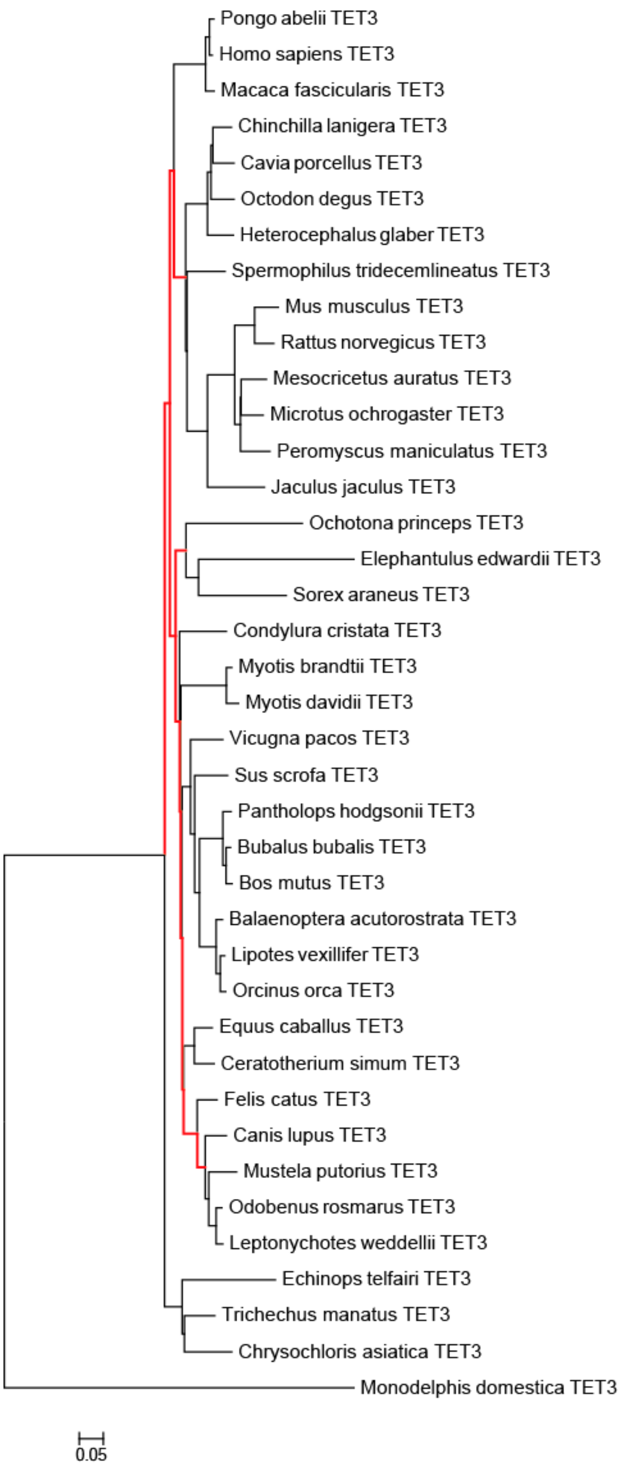

Figure S3. Maximum-likelihood tree of the TET3 genes. The tree was constructed using PhyML v3.0 software, based on the nucleotide-coding sequences. Branches with the number of sites under relaxed selection (ω_3 = 1.07) greater than the 90th percentile in the Fitmodel analysis (Figure S4) are shown in red.

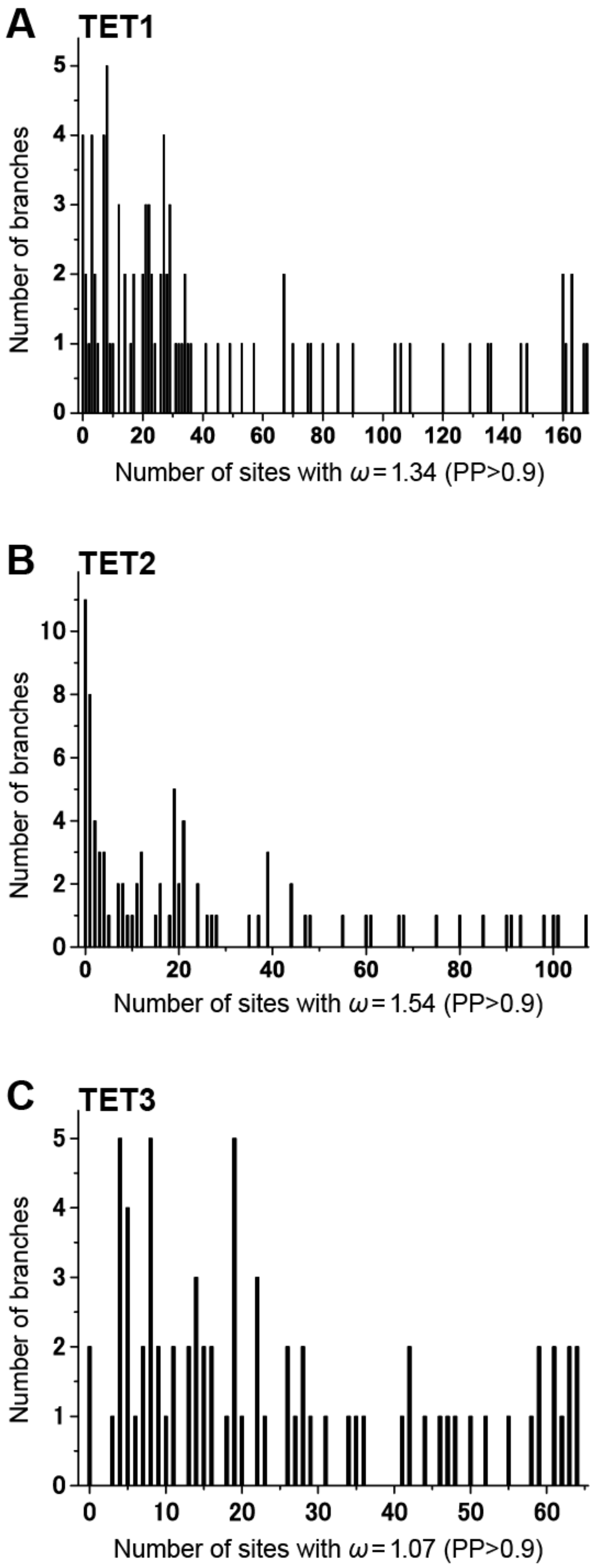

Figure S4. Distribution of branches with different numbers of sites under the ω_3 rate ratio class across TET1 (**A**), TET2 (**B**) and TET3 (**C**) gene trees. The *x*-axis represents the number of sites with the $\omega3$ rate ratio class; the *y*-axis represents the number of branches.

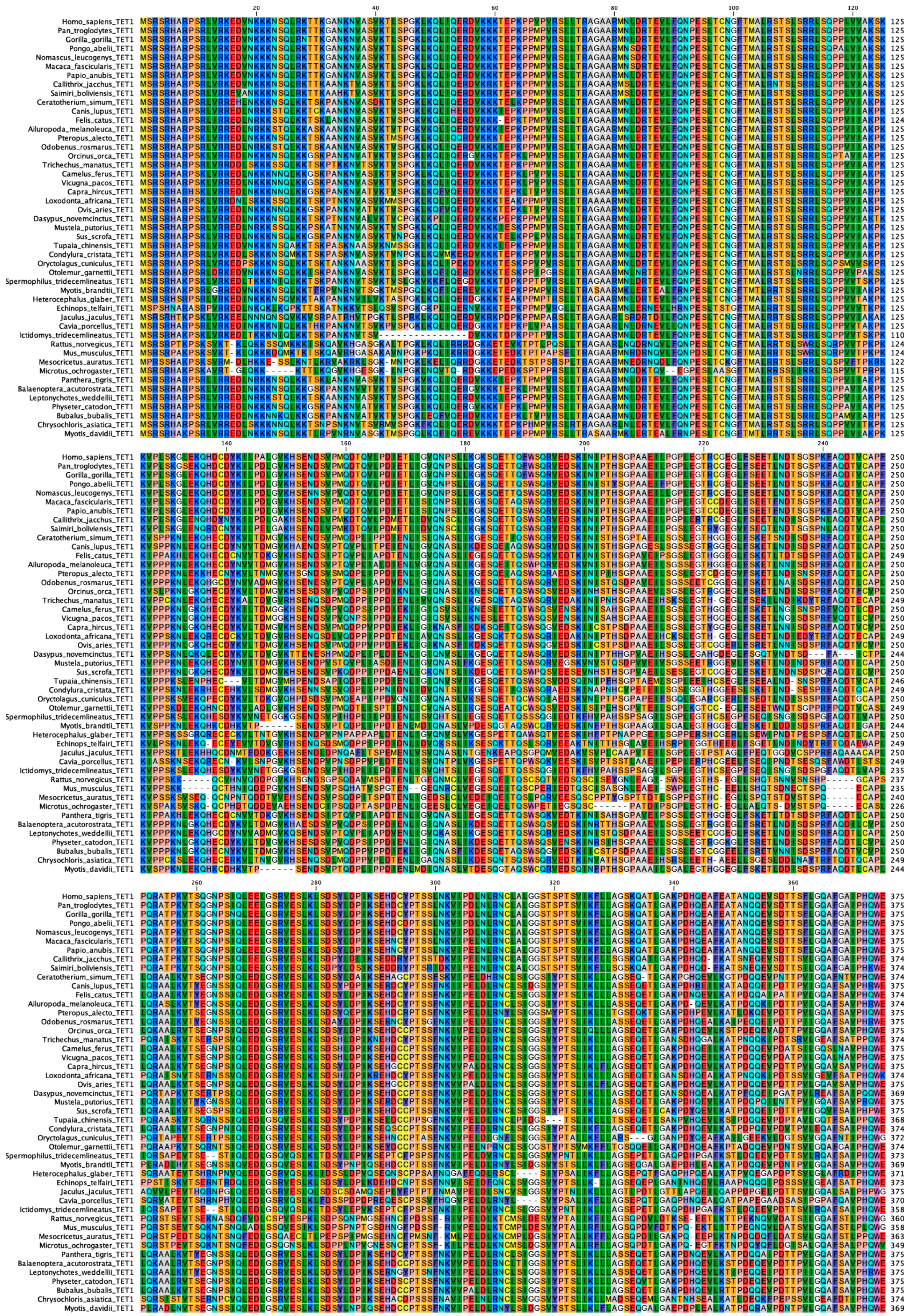

Figure S5. *Cont.*

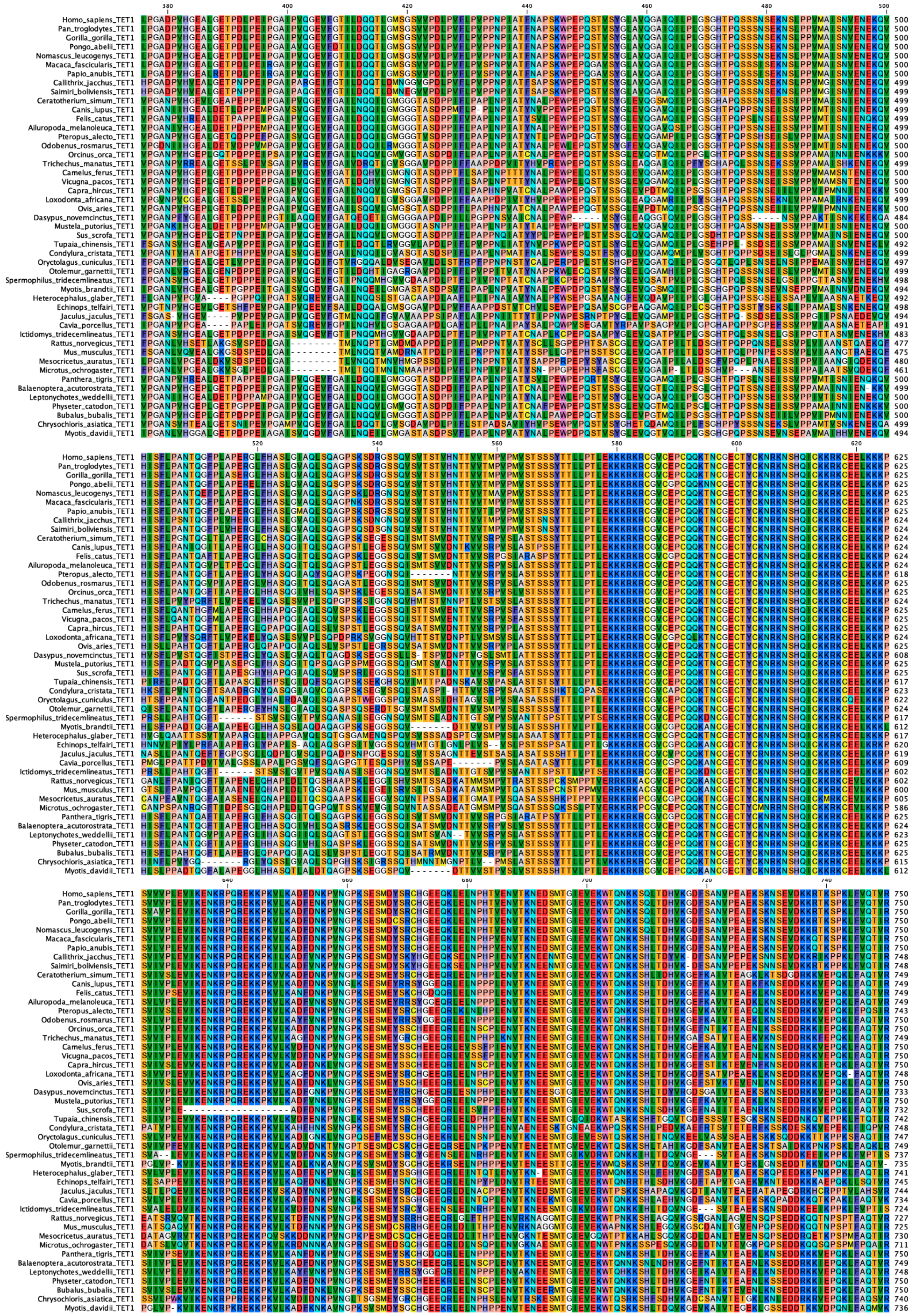

Figure S5. *Cont.*

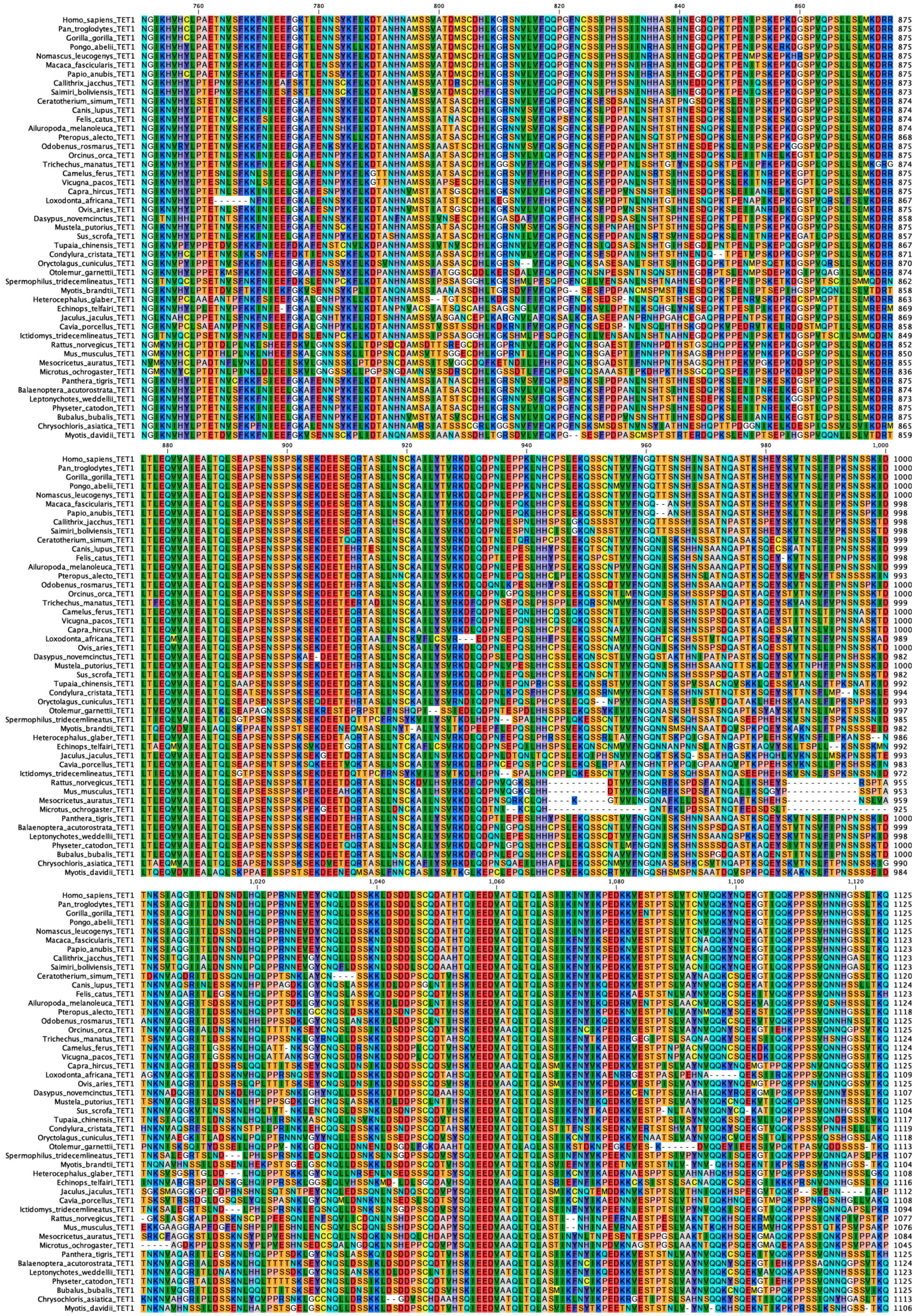

Figure S5. *Cont.*

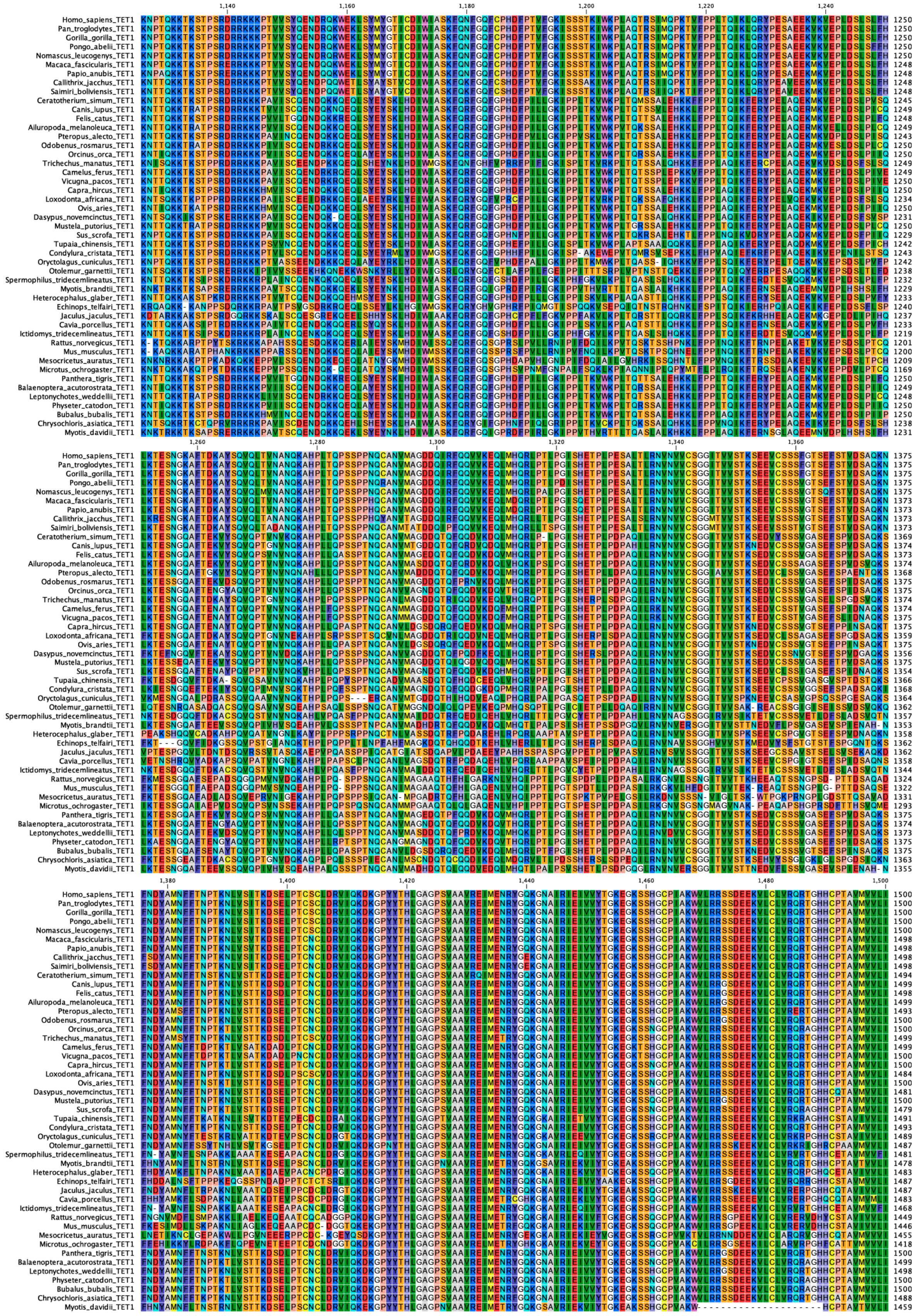

Figure S5. *Cont.*

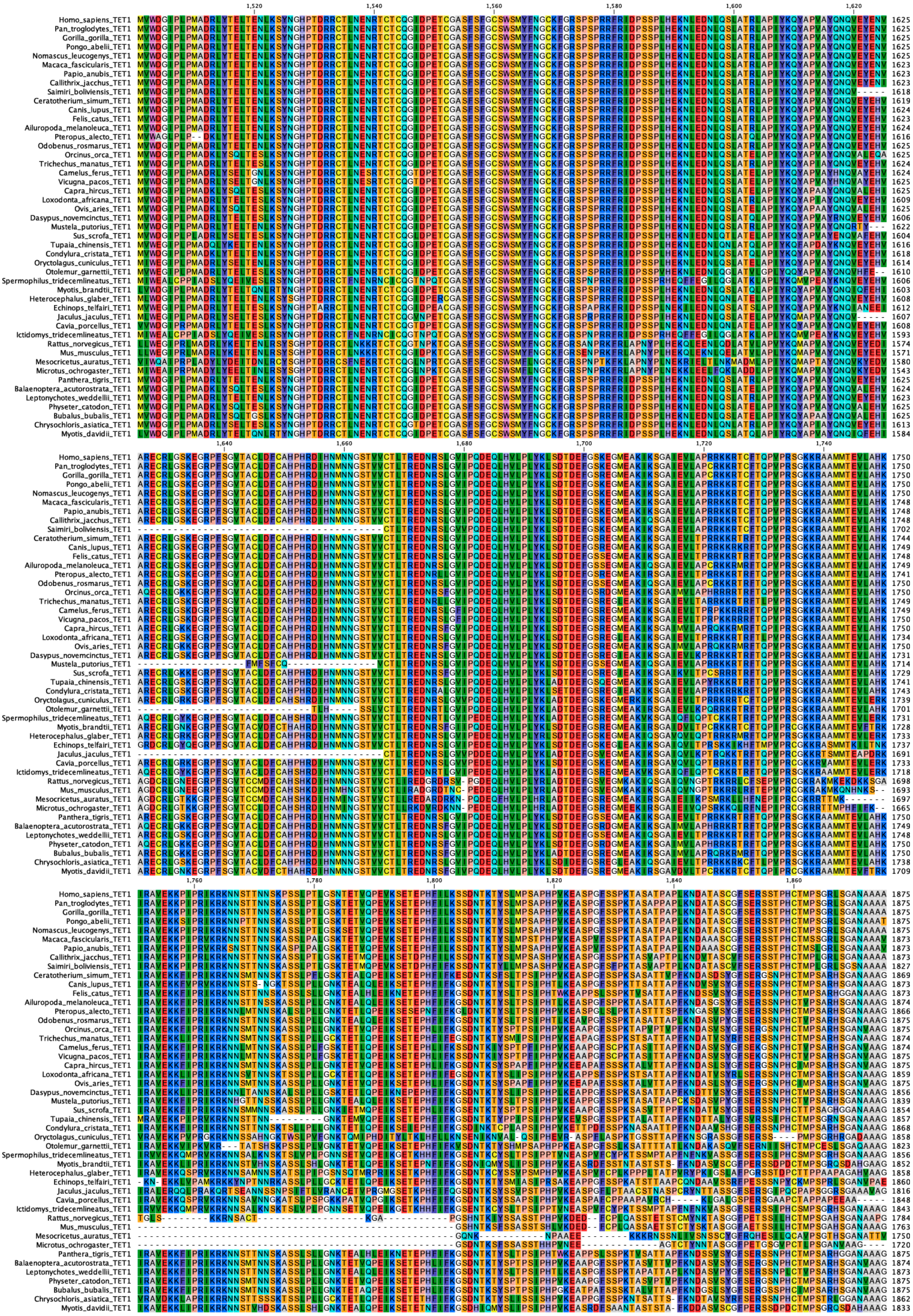

Figure S5. *Cont.*

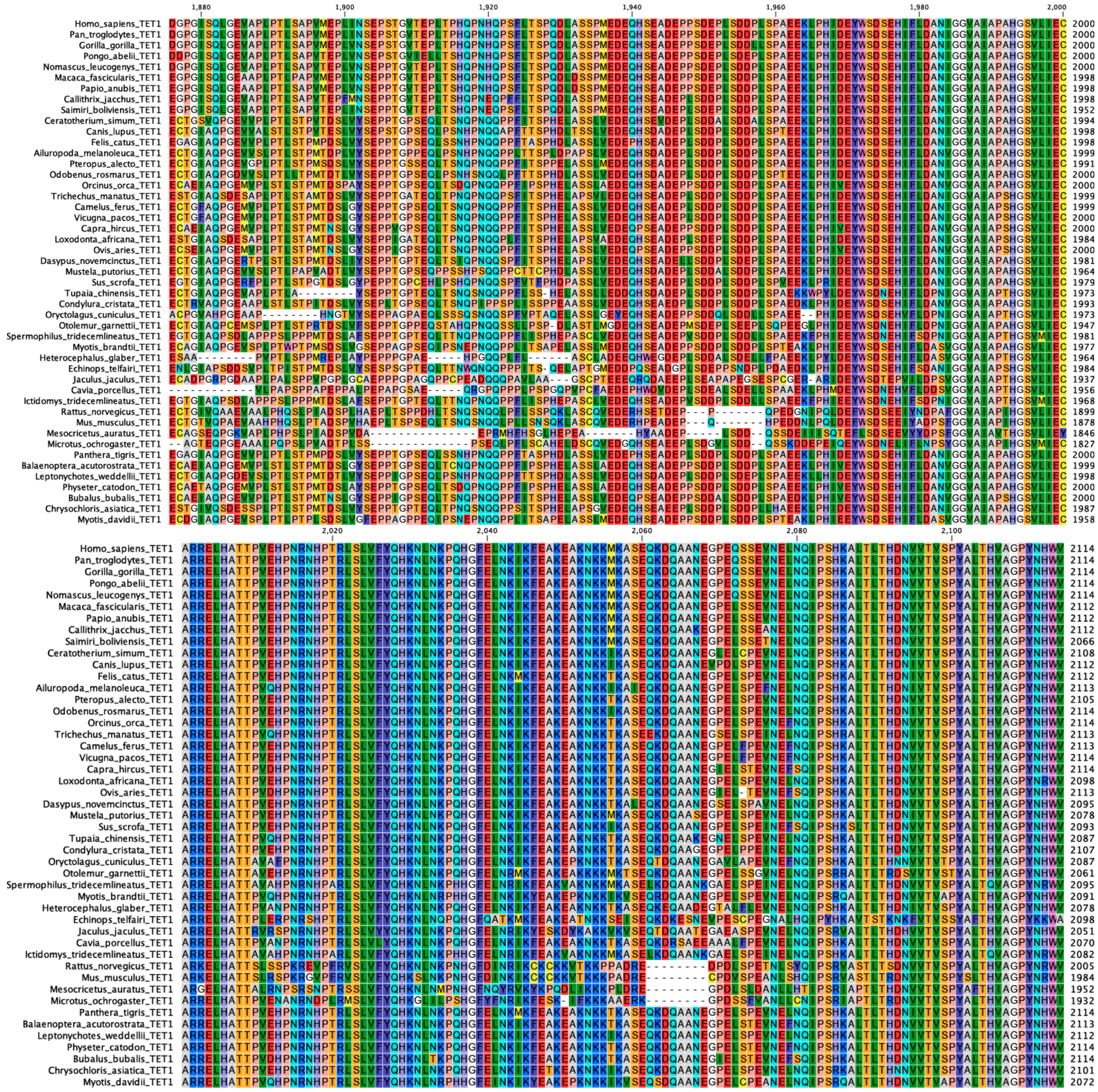

Figure S5. Multiple amino acid sequence alignment of mammalian TET1 genes. Amino acid sequences were aligned using MAFFT software with parameter settings optimized for the iterative refinement method (FFT-NS-i). The resulting multiple sequence alignment was trimmed by removing poorly aligned regions, using trimAl 1.2.

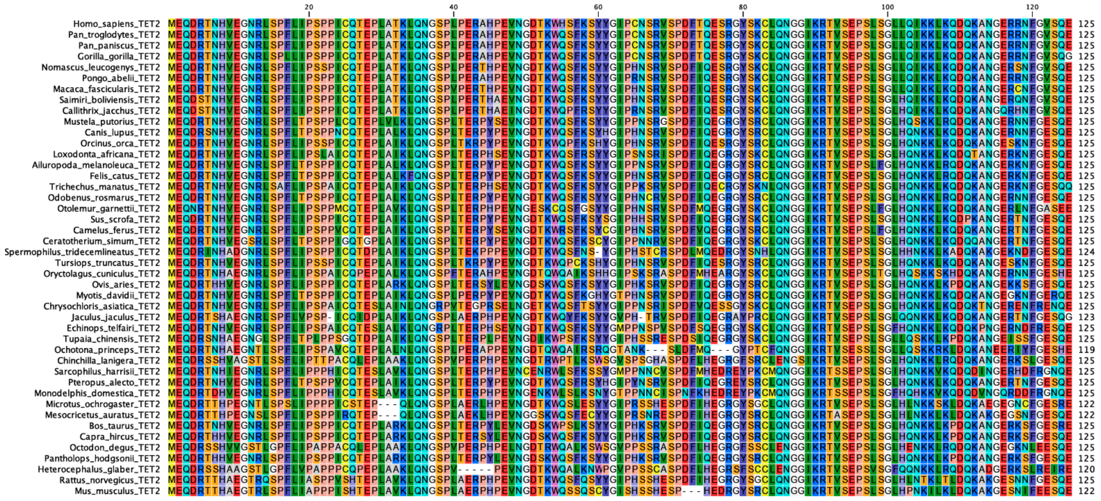

Figure S6. *Cont.*

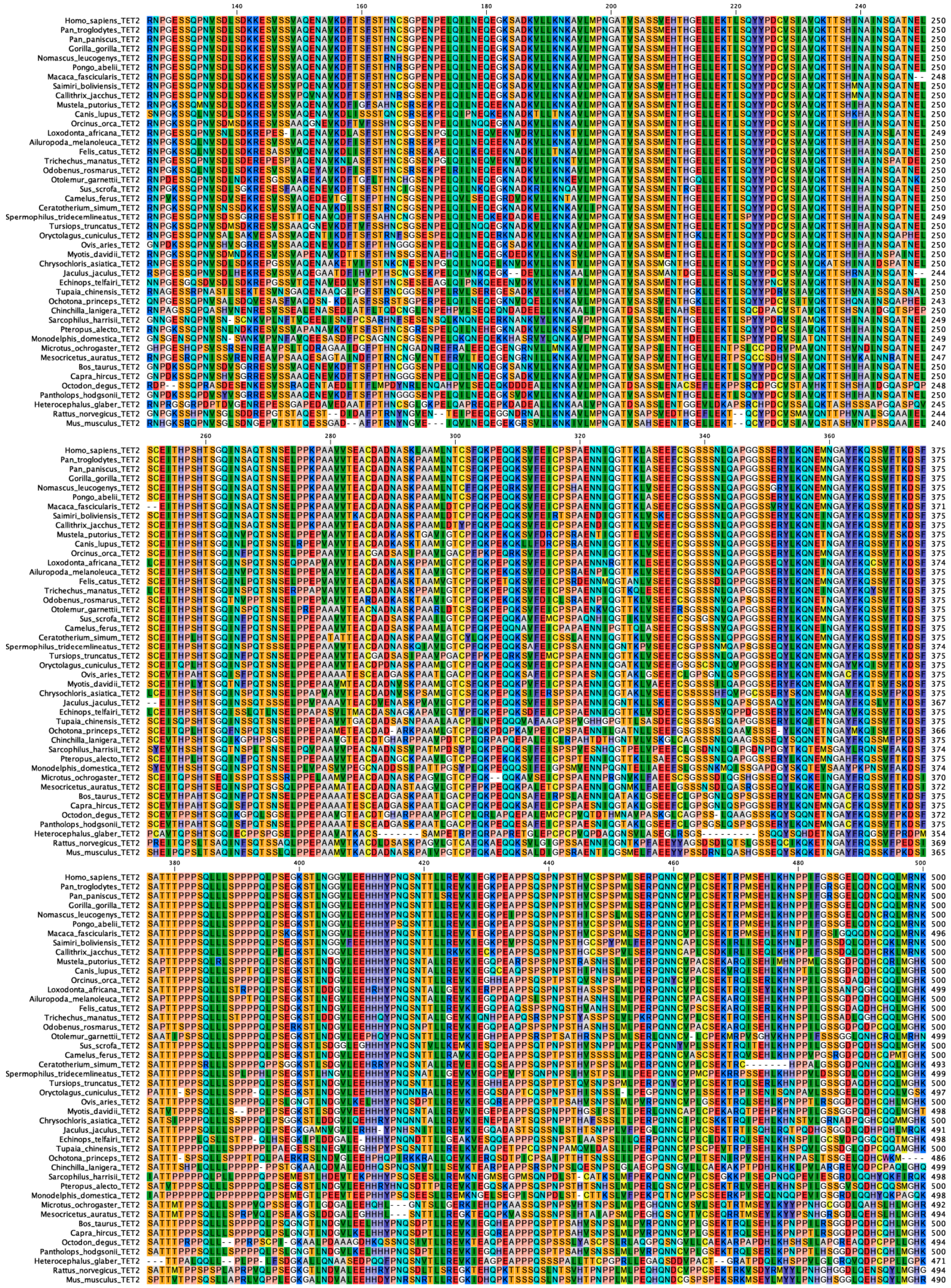

Figure S6. *Cont.*

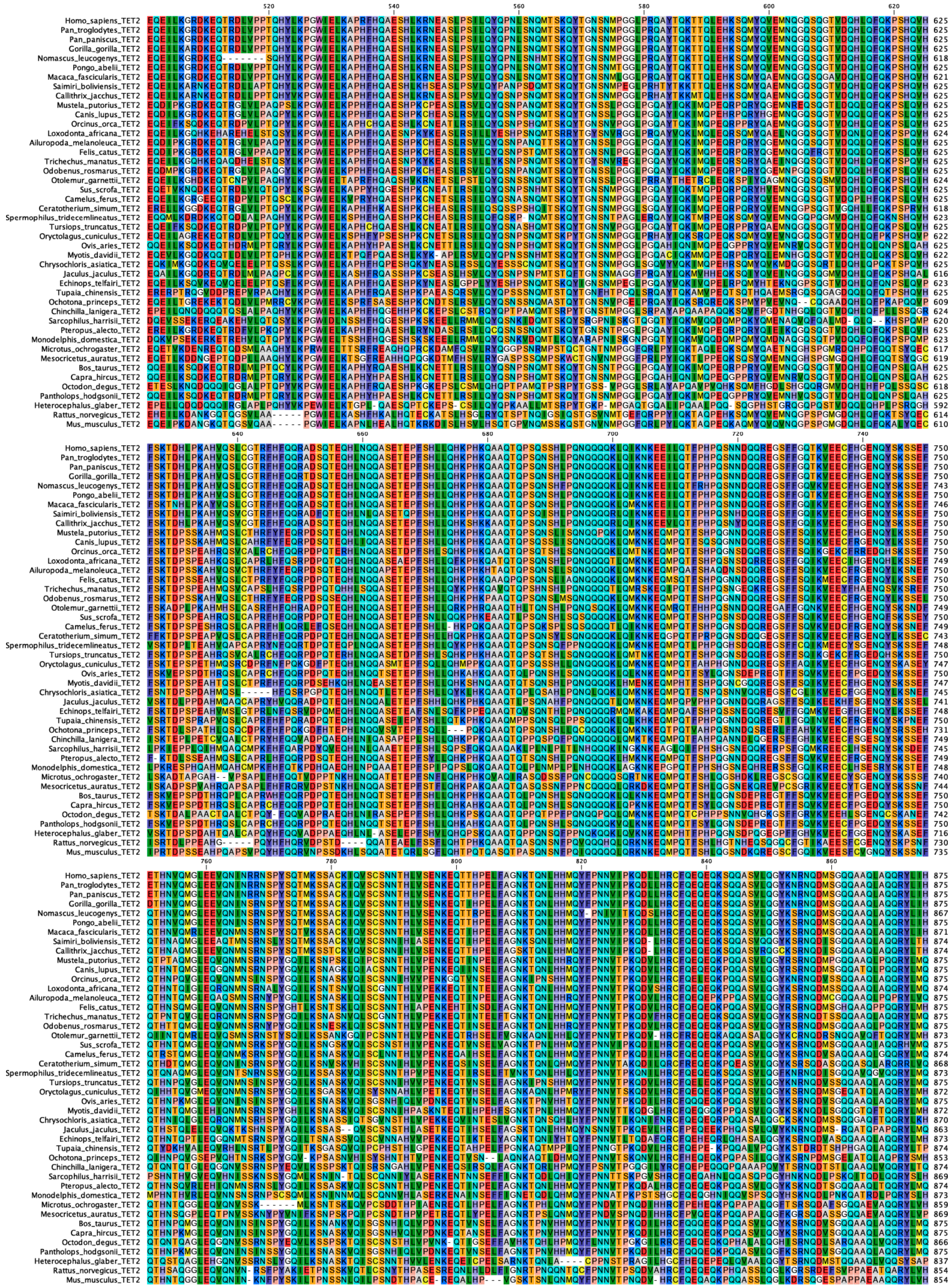

Figure S6. *Cont.*

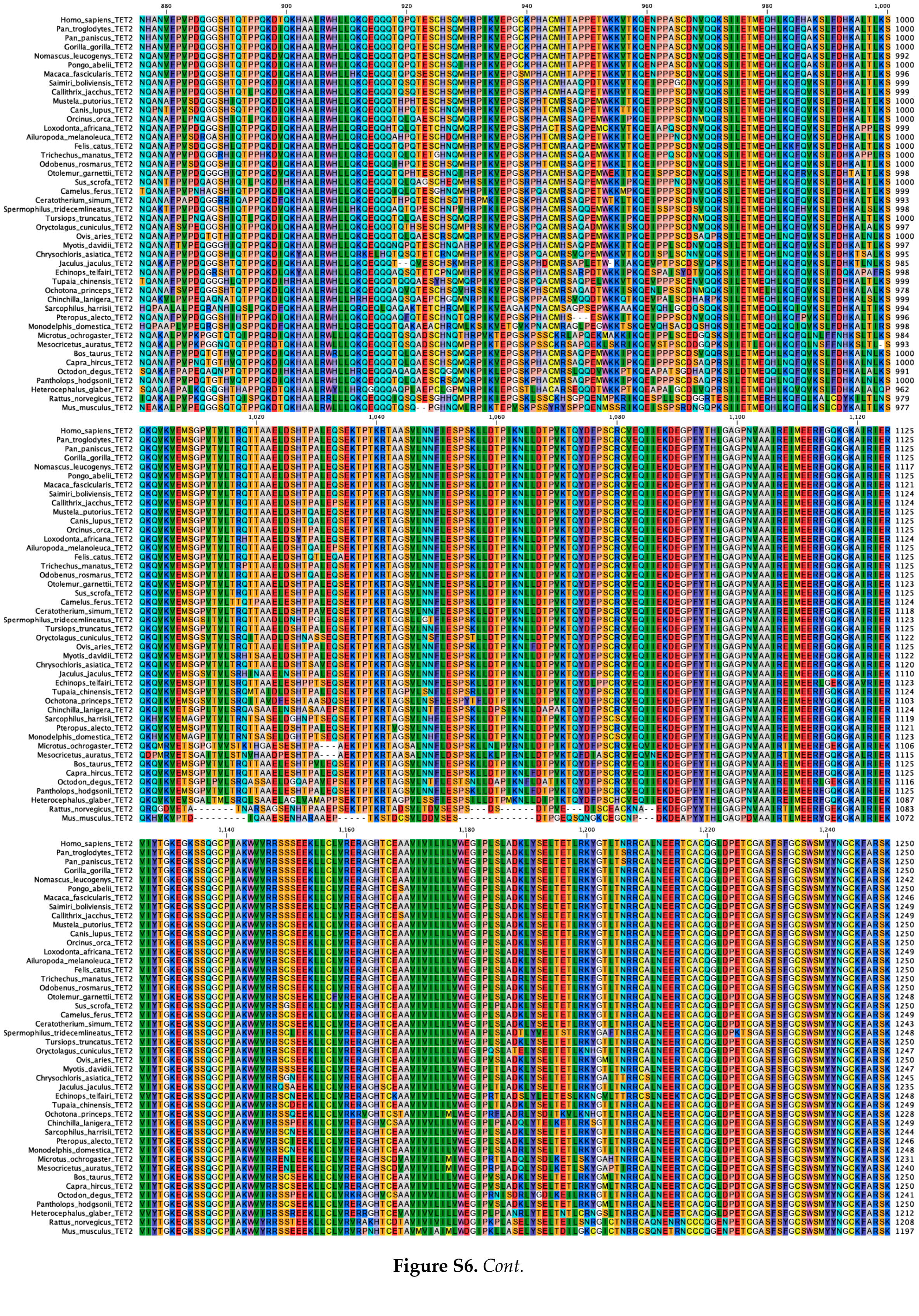

Figure S6. *Cont.*

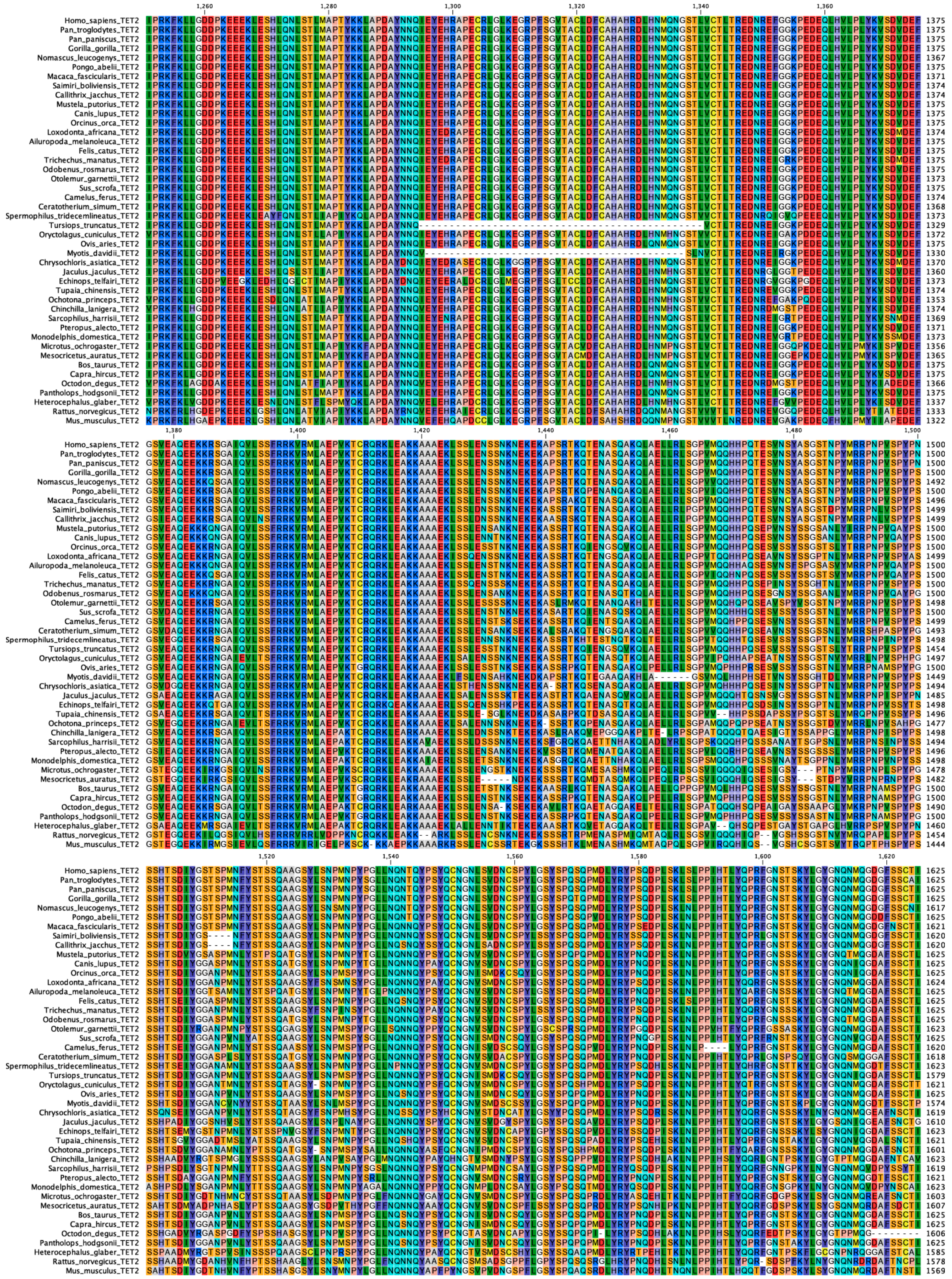

Figure S6. *Cont.*

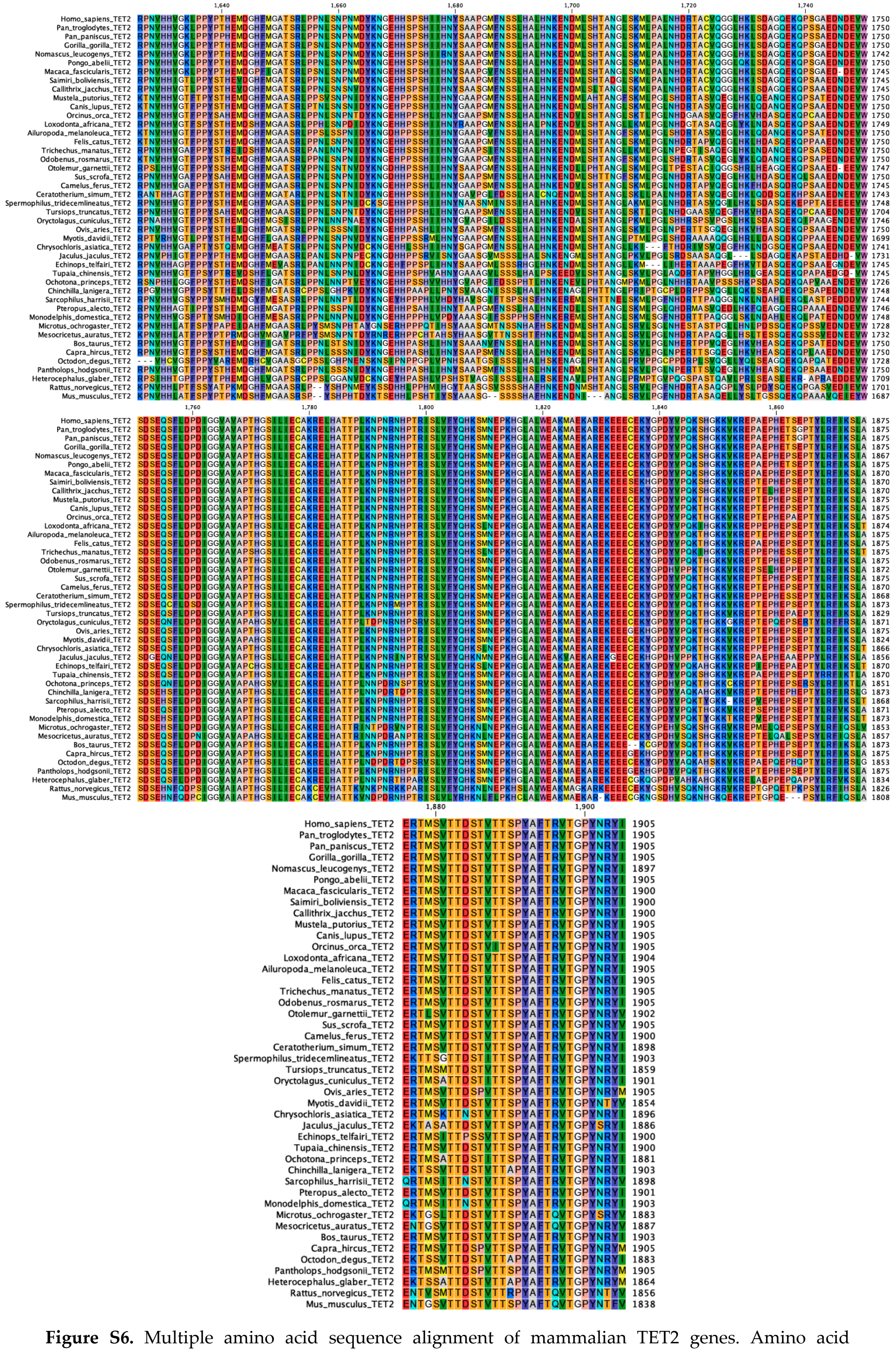

Figure S6. Multiple amino acid sequence alignment of mammalian TET2 genes. Amino acid sequences were aligned using MAFFT software with parameter settings optimized for FFT-NS-i. The resulting multiple sequence alignment was trimmed by removing poorly aligned regions, using trimAl 1.2.

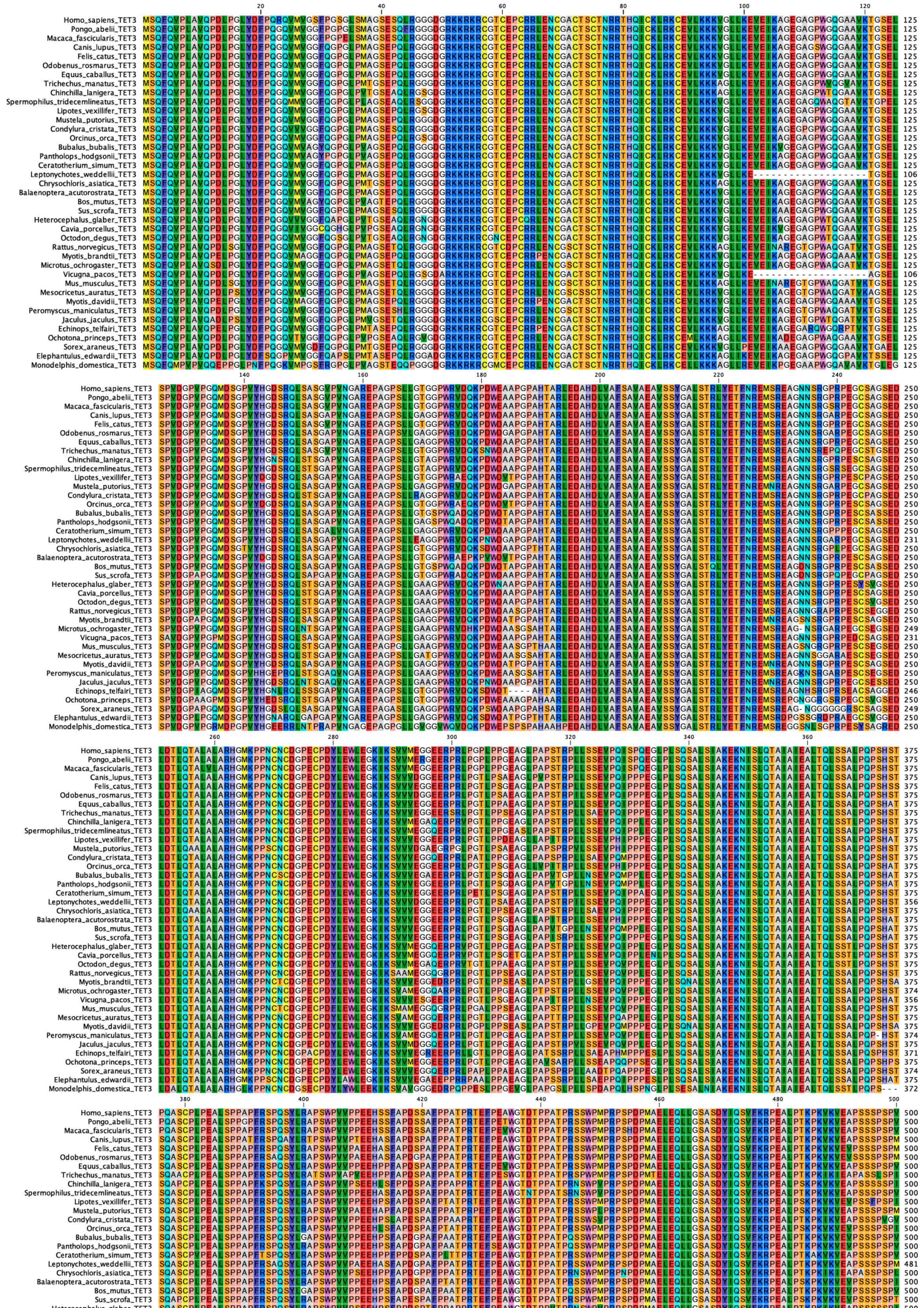

Figure S7. *Cont.*

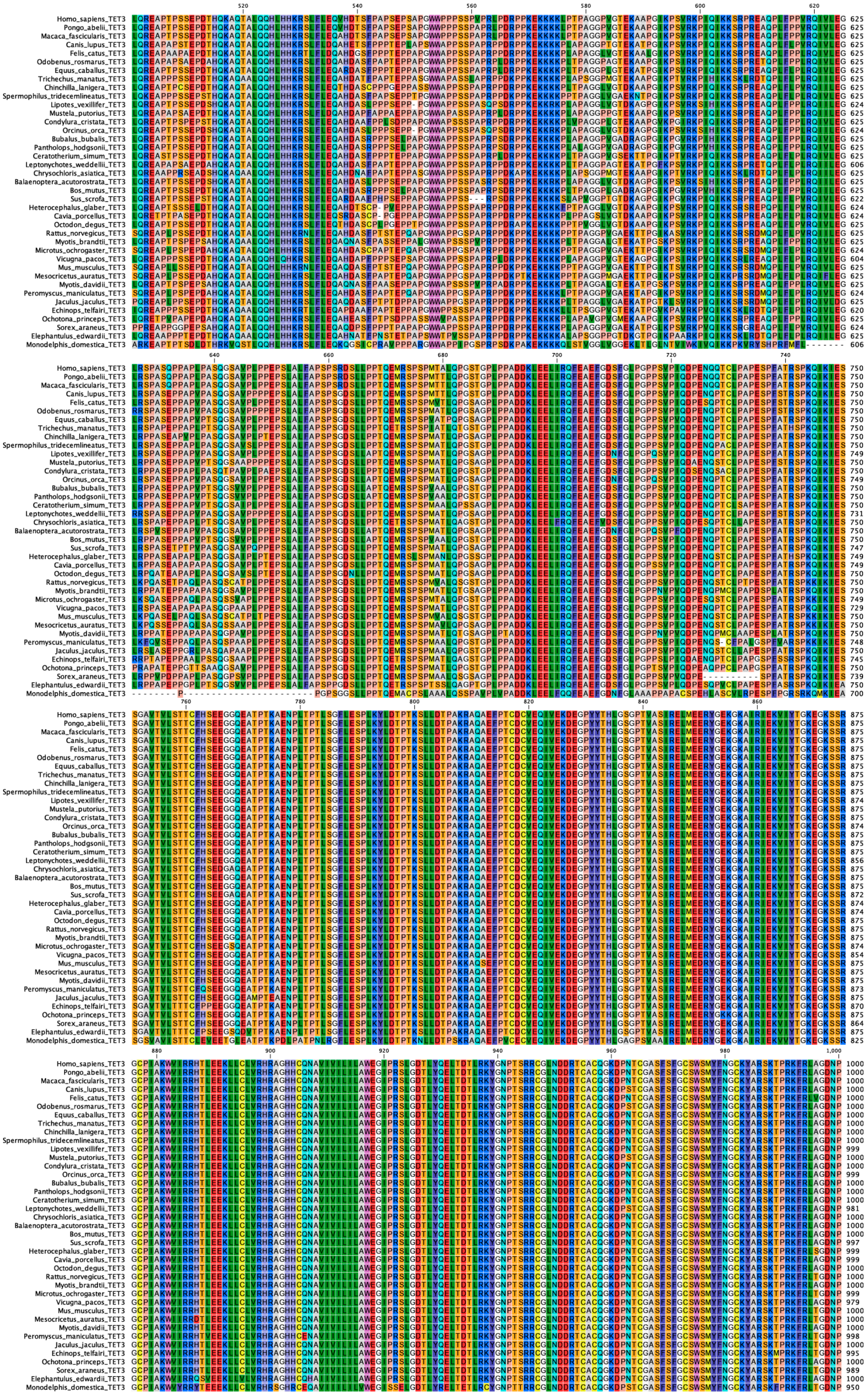

Figure S7. *Cont.*

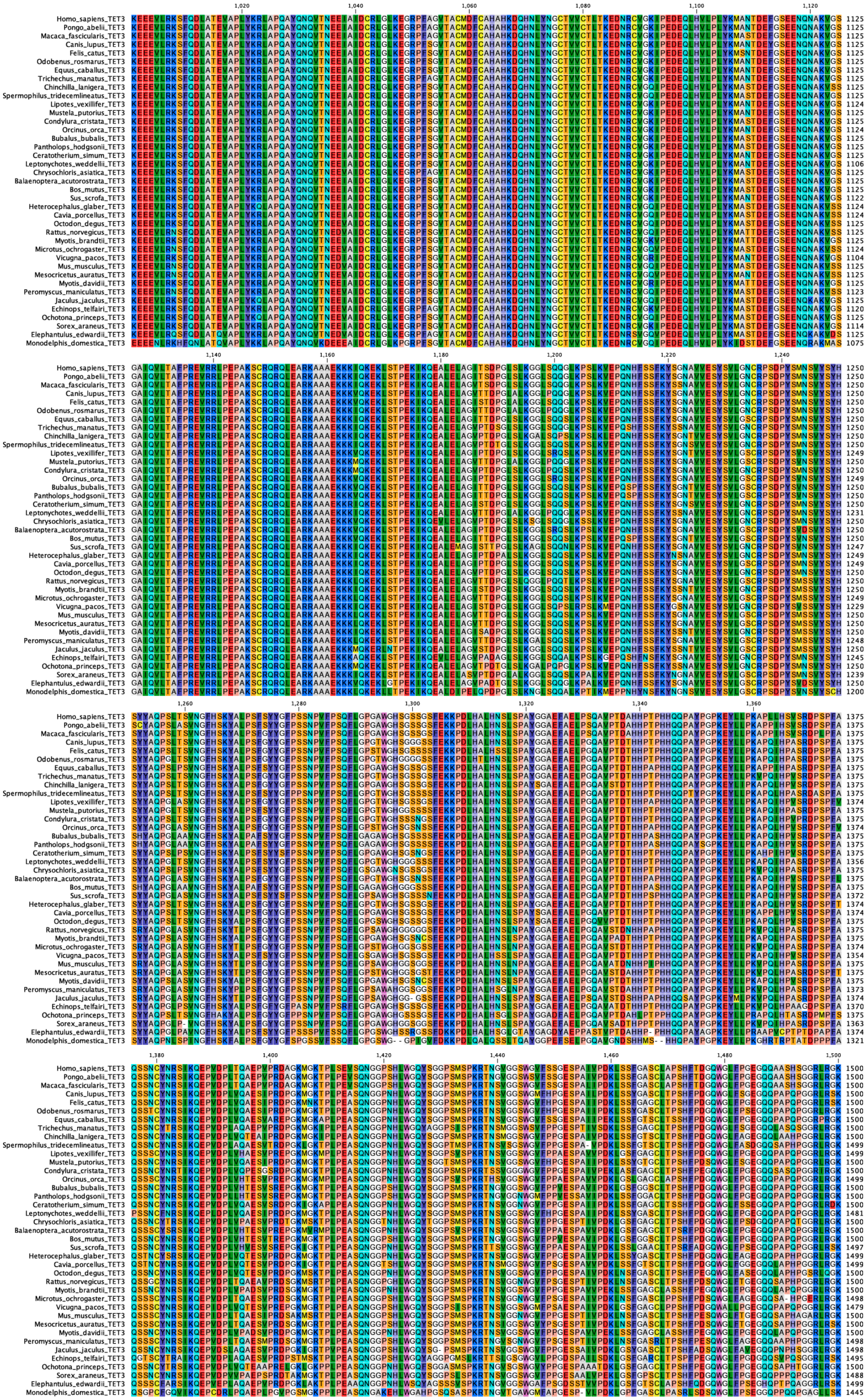

Figure S7. *Cont.*

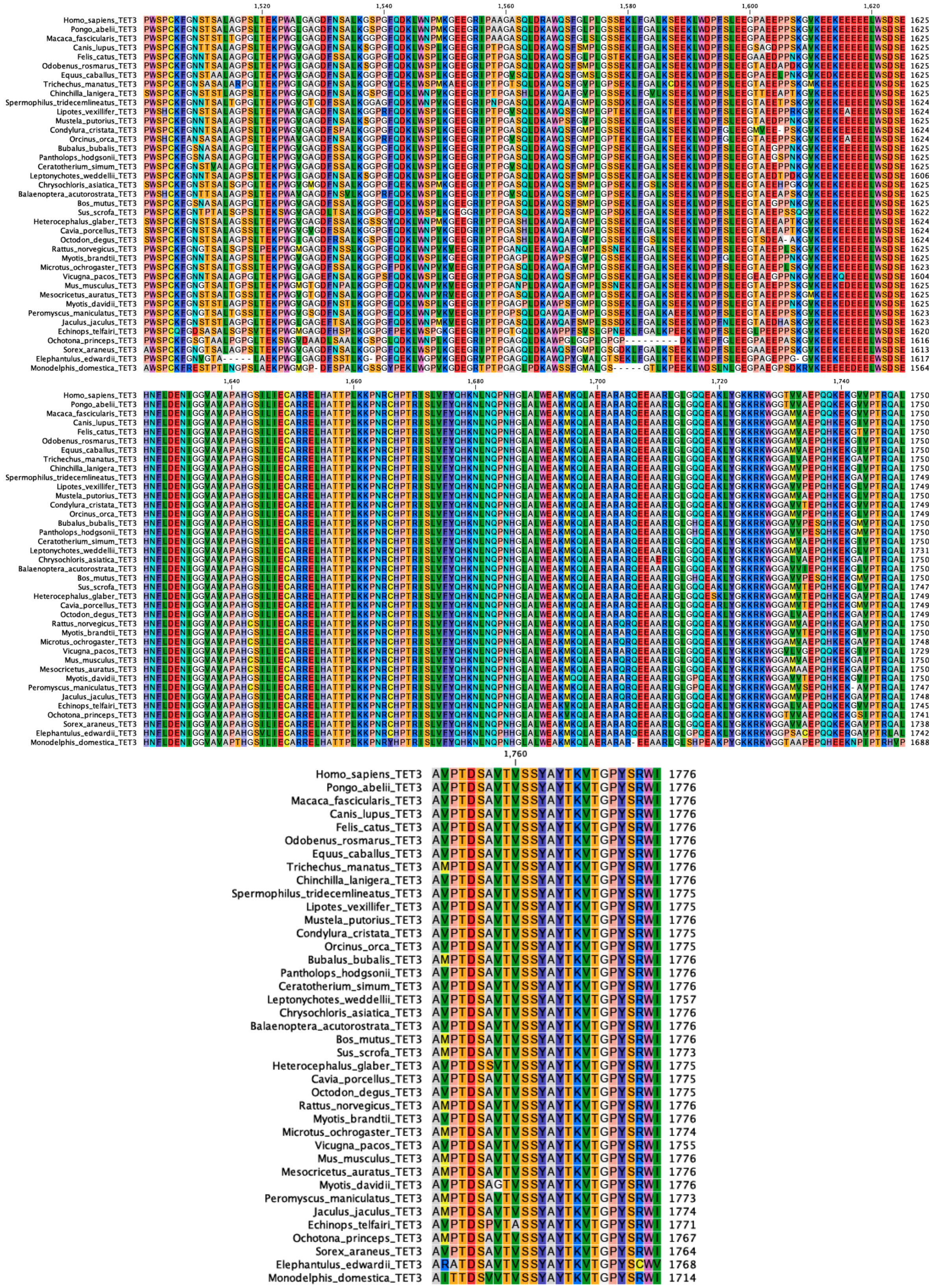

Figure S7. Multiple amino acid sequence alignment of mammalian TET3 genes. Amino acid sequences were aligned using MAFFT software with parameter settings optimized for FFT-NS-i. The resulting multiple sequence alignment was trimmed by removing poorly aligned regions, using trimAl 1.2.

Table S1. The TET genes used in this study.

Gene Name	Accession Number
Homo_sapiens_TET1	NM_030625
Pan_troglodytes_TET1	XM_507822
Gorilla_gorilla_TET1	XM_004049504
Pongo_abelii_TET1	XM_002820885
Nomascus_leucogenys_TET1	ENSNLEG00000011782
Macaca_fascicularis_TET1	XM_005565691
Papio_anubis_TET1	XM_003903859
Callithrix_jacchus_TET1	XM_002756304
Saimiri_boliviensis_TET1	XM_003928779
Ceratotherium_simum_TET1	XM_004427056
Canis_lupus_TET1	XM_536371
Felis_catus_TET1	ENSFCAG00000012673
Ailuropoda_melanoleuca_TET1	ENSAMEG00000018009
Pteropus_alecto_TET1	KB030800
Odobenus_rosmarus_TET1	XM_004393695
Orcinus_orca_TET1	XM_004280811
Trichechus_manatus_TET1	XM_004386395
Camelus_ferus_TET1	XM_006181184
Vicugna_pacos_TET1	XM_006212904
Capra_hircus_TET1	XM_005699119
Loxodonta_africana_TET1	XM_003409297
Ovis_aries_TET1	XM_004021627
Dasypus_novemcinctus_TET1	XM_004470524
Mustela_putorius_TET1	XM_004795559
Sus_scrofa_TET1	KC137683.1
Tupaia_chinensis_TET1	XM_006148936
Condylura_cristata_TET1	XM_004680833
Oryctolagus_cuniculus_TET1	XM_002718452
Otolemur_garnettii_TET1	XM_003783761
Spermophilus_tridecemlineatus_TET1	XM_005325968
Myotis_brandtii_TET1	XM_005876807
Heterocephalus_glaber_TET1	XM_004888511
Echinops_telfairi_TET1	XM_004701383
Jaculus_jaculus_TET1	XM_004657793
Cavia_porcellus_TET1	XM_003473584
Ictidomys_tridecemlineatus_TET1	ENSSTOG00000001527
Rattus_norvegicus_TET1	XM_006223879
Mus_musculus_TET1	NM_001253857
Mesocricetus_auratus_TET1	XM_005070904
Microtus_ochrogaster_TET1	XM_005360115
Panthera_tigris_TET1	XM_007095756
Balaenoptera_acutorostrata_TET1	XM_007167787
Leptonychotes_weddellii_TET1	XM_006728898
Physeter_catodon_TET1	XM_007121056
Bubalus_bubalis_TET1	XM_006070286
Chrysochloris_asiatica_TET1	XM_006835832
Myotis_davidii_TET1	XM_006760999
Homo_sapiens_TET2	NM_001127208
Pan_troglodytes_TET2	XM_003310400

Table S1. *Cont.*

Gene Name	Accession Number
Pan_paniscus_TET2	XM_003829967
Gorilla_gorilla_TET2	XM_004040224
Nomascus_leucogenys_TET2	ENSNLEG00000013289
Pongo_abelii_TET2	XM_002815025
Macaca_fascicularis_TET2	XM_005555594
Saimiri_boliviensis_TET2	XM_003929494
Callithrix_jacchus_TET2	XM_002745526
Mustela_putorius_TET2	XM_004748137
Canis_lupus_TET2	XM_535678
Orcinus_orca_TET2	XM_004269617
Loxodonta_africana_TET2	XM_003410366
Ailuropoda_melanoleuca_TET2	XM_002926670
Felis_catus_TET2	XM_006930937
Trichechus_manatus_TET2	XM_004380202
Odobenus_rosmarus_TET2	XM_004401875
Otolemur_garnettii_TET2	XM_003796334
Sus_scrofa_TET2	XM_003129278
Camelus_ferus_TET2	XM_006184616
Ceratotherium_simum_TET2	XM_004426613
Spermophilus_tridecemlineatus_TET2	XM_005339807
Tursiops_truncatus_TET2	XM_004319793
Oryctolagus_cuniculus_TET2	XM_002717196
Ovis_aries_TET2	XM_004009655
Myotis_davidii_TET2	XM_006778911
Chrysochloris_asiatica_TET2	XM_006874557
Jaculus_jaculus_TET2	XM_004662993
Echinops_telfairi_TET2	XM_004703292
Tupaia_chinensis_TET2	XM_006151157
Ochotona_princeps_TET2	XM_004594282
Chinchilla_lanigera_TET2	XM_005406228
Sarcophilus_harrisii_TET2	XM_003772926
Pteropus_alecto_TET2	KB031030
Monodelphis_domestica_TET2	XM_003341383
Microtus_ochrogaster_TET2	XM_005357312
Mesocricetus_auratus_TET2	XM_005081981
Bos_taurus_TET2	XM_001790146
Capra_hircus_TET2	XM_005681343
Octodon_degus_TET2	XM_004626707
Pantholops_hodgsonii_TET2	XM_005978869
Heterocephalus_glaber_TET2	XM_004866253
Rattus_norvegicus_TET2	XM_001077411
Mus_musculus_TET2	NM_001040400
Homo_sapiens_TET3	NM_001287491
Pongo_abelii_TET3	ENSPPYG00000012286
Macaca_fascicularis_TET3	XM_005575570
Canis_lupus_TET3	XM_005630554
Felis_catus_TET3	XM_003984133
Odobenus_rosmarus_TET3	XM_004398707
Equus_caballus_TET3	XM_001917114
Trichechus_manatus_TET3	XM_004369189

Table S1. *Cont.*

Gene Name	Accession Number
Chinchilla_lanigera_TET3	XM_005385530
Spermophilus_tridecemlineatus_TET3	XM_005329351
Lipotes_vexillifer_TET3	XM_007459392
Mustela_putorius_TET3	XM_004742217
Condylura_cristata_TET3	XM_004691540
Orcinus_orca_TET3	XM_004277087
Bubalus_bubalis_TET3	XM_006045954
Pantholops_hodgsonii_TET3	XM_005962644
Ceratotherium_simum_TET3	XM_004435517
Leptonychotes_weddellii_TET3	XM_006735303
Chrysochloris_asiatica_TET3	XM_006872404
Balaenoptera_acutorostrata_TET3	XM_007189011
Bos_mutus_TET3	XM_005897644
Sus_scrofa_TET3	XM_005662450
Heterocephalus_glaber_TET3	XM_004844710
Cavia_porcellus_TET3	XM_005003343
Octodon_degus_TET3	XM_004634101
Rattus_norvegicus_TET3	XM_006224966
Myotis_brandtii_TET3	XM_005869422
Microtus_ochrogaster_TET3	XM_005364657
Vicugna_pacos_TET3	XM_006218996
Mus_musculus_TET3	XM_006505773
Mesocricetus_auratus_TET3	XM_005071053
Myotis_davidii_TET3	XM_006760024
Peromyscus_maniculatus_TET3	XM_006994349
Jaculus_jaculus_TET3	XM_004668275
Echinops_telfairi_TET3	XM_004696098
Ochotona_princeps_TET3	XM_004590675
Sorex_araneus_TET3	XM_004619988
Elephantulus_edwardii_TET3	XM_006880851
Monodelphis_TET3	XM_007477889
